# An Integrative Approach for Modeling and Simulation of Heterocyst Pattern Formation in Cyanobacteria Filaments

**DOI:** 10.1371/journal.pcbi.1004129

**Published:** 2015-03-27

**Authors:** Alejandro Torres-Sánchez, Jesús Gómez-Gardeñes, Fernando Falo

**Affiliations:** 1 Departamento de Física de la Materia Condensada, Universidad de Zaragoza, Zaragoza, Spain; 2 Laboratori de Càlcul Numèric, Universitat de Politècnica de Catalunya, Barcelona, Spain; 3 Instituto de Biocomputación y Física de Sistemas Complejos (BIFI), Universidad de Zaragoza, Zaragoza, Spain; Princeton University, UNITED STATES

## Abstract

Heterocyst differentiation in cyanobacteria filaments is one of the simplest examples of cellular differentiation and pattern formation in multicellular organisms. Despite of the many experimental studies addressing the evolution and sustainment of heterocyst patterns and the knowledge of the genetic circuit underlying the behavior of single cyanobacterium under nitrogen deprivation, there is still a theoretical gap connecting these two macroscopic and microscopic processes. As an attempt to shed light on this issue, here we explore heterocyst differentiation under the paradigm of systems biology. This framework allows us to formulate the essential dynamical ingredients of the genetic circuit of a single cyanobacterium into a set of differential equations describing the time evolution of the concentrations of the relevant molecular products. As a result, we are able to study the behavior of a single cyanobacterium under different external conditions, emulating nitrogen deprivation, and simulate the dynamics of cyanobacteria filaments by coupling their respective genetic circuits via molecular diffusion. These two ingredients allow us to understand the principles by which heterocyst patterns can be generated and sustained. In particular, our results point out that, by including both diffusion and noisy external conditions in the computational model, it is possible to reproduce the main features of the formation and sustainment of heterocyst patterns in cyanobacteria filaments as observed experimentally. Finally, we discuss the validity and possible improvements of the model.

## Author Summary

Cyanobacteria filaments are paradigmatic examples of prokaryotic cellular differentiation and cooperative pattern formation. When a cyanobacteria filament is deprived of combined nitrogen, some vegetative cells differentiate into heterocysts, which are terminally differentiated nitrogen-fixing cells. Interestingly, most cells do not differentiate, but remain in their initial vegetative state. The coexistence of heterocysts and vegetative cells is essential for the survival of the filament since (*i*) heterocysts lose their photosynthetic capacity so they need vegetative cells around to be provided with a source of fixed carbon and (*ii*) cell division, i.e. reproduction, is only accomplished by vegetative cells. From such a paradigmatic example it is clear that differentiation processes are the result of the interplay of complex regulatory networks acting inside the cell and external stimuli, coming from both the adjacent cells and the environment. In this article we present an integrative approach that combines the study of internal regulatory processes, diffusion, and noisy environments in order to capture the key mechanisms leading to the differentiation of vegetative cyanobacteria into heterocysts and the subsequent pattern formation.

## Introduction

The formation of multicellular organisms from the assembly of single-celled ones constitutes one of the most striking and complex problems tackled by biology. The most salient feature that characterizes multicellular organisms is the presence of different cell types, in such a way that the organism associates a different function to each cell type. In each of these cellular types, only a subset of the genes that constitute the genome of the organism (genotype) are expressed, which identify the function and morphology of the cell (phenotype). The development of specialized cells involves differentiation processes, which lead to alterations in gene expression producing different phenotypes from a given genotype. These processes are highly dynamical, directed by complex regulatory networks involving cell-to-cell interactions, and often triggered by external stimuli. As a result of the differentiation processes a rich cooperative pattern involving different cell types is established, increasing the complexity and adaptability of the organism. Due to the large number of scales involved, ranging from protein binding to diffusion of specific elements throughout the organism, a correct mathematical modeling of differentiation processes and their associated pattern formation demands an integrative approach combining tools from statistical mechanics and the theory of dynamical systems (see [1, 2] for instance).

A landmark process of (prokaryotic) cellular differentiation and cooperative pattern formation is the heterocyst differentiation in cyanobacteria filaments [3, 4]. Cyanobacteria are one of the first organisms that developed multicellularity some (2–3) billion years ago [5]. These bacteria perform oxygenic photosynthesis releasing oxygen to the environment. However, nitrogenase, the enzyme that performs nitrogen fixation, is deactivated by oxygen so that nitrogen fixation cannot occur in its presence [6]. Cyanobacteria solve the incompatibility of incorporating both oxygenic photosynthesis and nitrogen fixation by separating these processes (*i*) temporally, such as in the unicellular *Cyanothece sp.* strain ATCC 51142, which presents photosynthetic activity during the day and fixes nitrogen during the night [7], or (*ii*) spatially, by the generation of non-photosynthetic nitrogen-fixing cells distributed along the filament and acting as nitrogen suppliers.

In the presence of combined nitrogen (such as nitrate, nitrite, ammonium or urea), most cyanobacteria (*Anabaena* PCC strain 7120 being the most representative example) form long filaments of photosynthetic vegetative cells. However, in the absence of combined nitrogen (cN), a subset of the vegetative cells differentiate into heterocysts, which are terminally differentiated nitrogen-fixing cells. By differentiating, heterocysts lose their photosynthetic capacity, so they require an external source of fixed carbon [8, 9]. To this aim, each forming heterocyst sends a signal, by means of the production of some substance that diffuses along the filament, to prevent the differentiation of its neighboring cells. A cooperative pattern is thus established: heterocysts provide cN to the filament while vegetative cells supply fixed carbon. As a result, heterocysts appear interspersed with around 10 vegetative cells, depending on the species, forming a semi-regular pattern that remains approximately constant regardless of cell division [10, 11]. The resulting pattern forms one of the simplest and most primitive examples of a multicellular organism as a product of the interdependence between heterocysts and vegetative cells. Interestingly, an isolated cyanobacterium does not differentiate but it first divides so that one of the descendants differentiates. This latter mechanism is crucial since (*i*) a sole heterocyst would lack a source of fixed carbon and (*ii*) it would not reproduce as it is a terminally differentiated cell [12].

Let us briefly review the previous studies on the mathematical modeling of heterocyst pattern formation. In references [13, 14] Rutenberg and coworkers analyzed a model to explain heterocyst patterns by means of the study of cN diffusion along a cyanobacterial filament. On the other hand, Gerdtzen *et al.* [15] modeled cyanobacterial filaments based on a time-discrete dynamical system incorporating the main interactions between the most important proteins that take part in heterocyst formation.

In this work, we develop a simple mathematical model by incorporating the recent experimental results on the genetic regulatory network of cyanobacteria into the theoretical machinery of system biology.

Our model connects the diffusion of combined nitrogen along the filament with the dynamical properties of the underlying genetic circuit of each single cyanobacterium, capturing both the development of heterocyst patterns and their maintenance. Furthermore, our model shows that noise plays an important role in the onset of differentiation by enabling the development of the characteristic heterocyst patterns for a wide range of model parameters. This reveals that cyanobacteria filaments have developed an efficient response to the noisy conditions that characterize the natural environment.

The work is structured as follows. First we present the main actors of the basic regulatory network and the different dynamical interactions that take place during the differentiation process. Then we develop a mathematical model for the unicellular reaction to nitrogen deprivation. Although a single cell model cannot provide a complete understanding of heterocyst formation, we analyze the main features that arise from the dynamical behavior of the system to gain insight about cell dynamics under different external conditions.

Finally, we round off the paper by introducing the spatial model consisting of a filament of cyanobacteria, each one characterized by the dynamical circuit developed previously, that interact by means of protein diffusion.

## Results

### Description of the main genes and their basic genetic circuit

**Fig 1:**
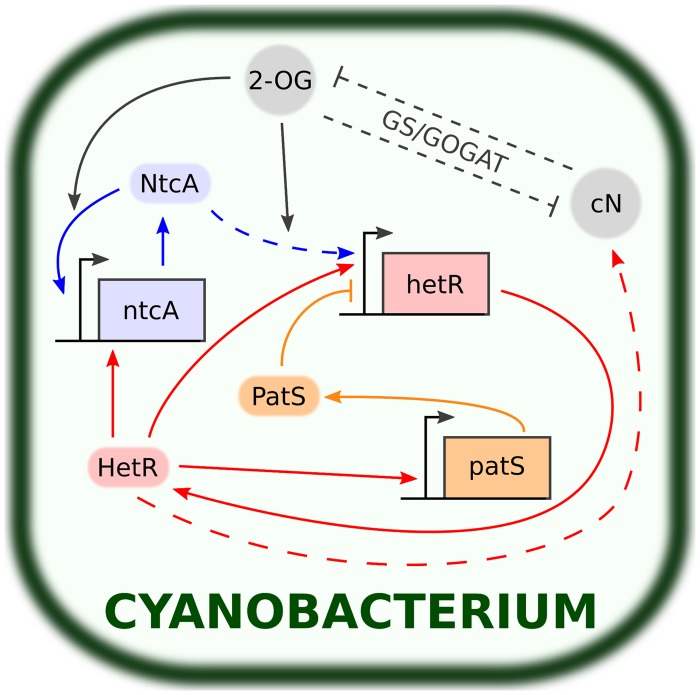
Main components and interactions involved in the reaction to combined nitrogen deprivation in cyanobacteria. Rectangular boxes represent genes (*ntcA, hetR* and *patS*) while rounded boxes and circles represent transcription factors (NtcA, HetR and PatS) and smaller molecules (2-OG and cN) respectively. Normal-tipped and flat-tipped arrows stand for up-regulating and down-regulating processes respectively. Dashed lines stand for indirect or imperfectly understood interactions. The accumulation of 2-OG enhances the DNA-binding activity of NtcA, which in turn up-regulates the transcription of *ntcA* and *hetR*. HetR activates *ntcA* and *hetR* (composing the central NtcA-HetR autoregulatory loop), the inhibitor *patS* and other genes that lead to nitrogen fixation and the morphological changes involved in heterocyst differentiation. 2-OG and cN levels are linked through the GS/GOGAT cycle (see Fig. 2).

Heterocyst development begins with sensing combined-nitrogen (cN) limitation and ends with nitrogen fixation in mature heterocysts. This process is usually completed after 20 hours at 30^∘^C [9]. In Fig. 1 we show a basic scheme of the genetic circuit including the most relevant elements and their respective interactions. Here we explain the main features of this genetic circuit.

**Fig 2:**
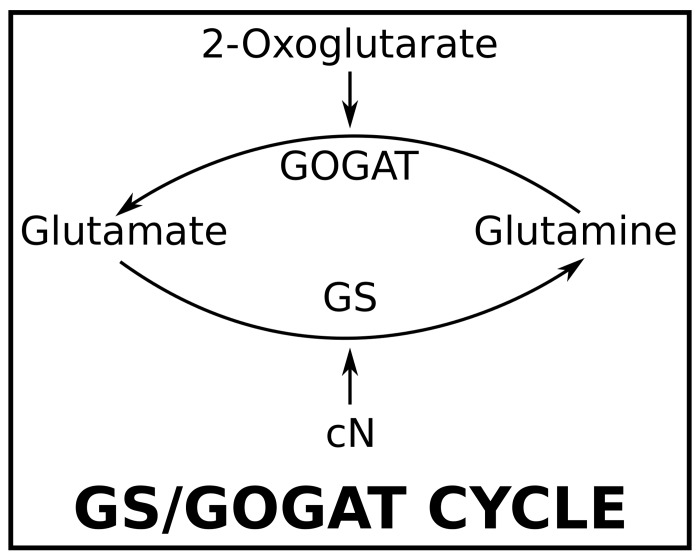
GS/GOGAT cycle. 2-OG and cN indirectly interact through the GS/GOGAT cycle. Glutamine is transformed into glutamate by means of 2-OG through the 2-OG amidotransferase (GOGAT) while cN converts glutamate into glutamine through the glutamine synthetase (GS). The importance of the cycle in heterocyst differentiation is twofold. From one side, it constitutes the early one-cell sensor to nitrogen starvation: the absence of cN breaks the cycle down and 2-OG starts to accumulate, whose action leads to the cascade of processes that provoke the differentiation (see Fig. 1). Additionally, later during the differentiation, it processes the cN created by the heterocysts decreasing the levels of 2-OG. The latter is crucial for the formation of the heterocyst pattern (see Fig. 3).

The process is initiated with the accumulation of 2-oxoglutarate (2-OG) as a consequence of cN deprivation [9, 16]. 2-OG interacts with ammonium through the GS/GOGAT cycle [17–19] (see Fig. 2). Under cN starvation, the GS/GOGAT cycle breaks down, leading to the accumulation of 2-OG inside the cell [9]. In its turn, 2-OG stimulates the DNA-binding activity of NtcA, an important transcription factor for heterocyst development [18, 20, 21]. Furthermore, the transcription of the genes targeted by NtcA does not start in the absence of 2-OG [22, 23].

NtcA presents autoregulation [22, 24, 25] and indirectly activates the key gene that controls cell differentiation and pattern formation: *hetR* [26–28]. To bind DNA, NtcA needs to homodimerize [29, 30]. In conclusion, the accumulation of 2-OG is the factor that triggers differentiation. In agreement with this idea, artificial increased levels of 2-OG result in heterocyst development even in the presence of ammonium [16, 18, 31].

The next step in heterocyst development is the activation of *hetR*. Remarkably, null mutants of *hetR* do not produce heterocysts whereas an overexpression of *hetR* leads to an increased heterocyst frequency [27, 32, 33]. The transcription of *hetR* is induced by NtcA through the action of an intermediate, *nrrA* [28]. The DNA-binding activity of HetR requires its homodimerization [34, 35]. Multiple transcription factors related to heterocyst formation are up-regulated by HetR, including *hetR* itself [35], *ntcA* [36] and *patS* [35].

**Fig 3:**
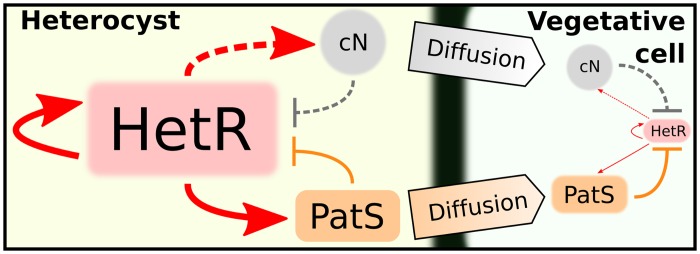
Diffusion scheme. Schematic representation of the diffusion processes that sustain the heterocyst pattern. Heterocysts produce cN and PatS. cN diffuses along the filament where, due to the action of the GS/GOGAT cycle (see Fig. 2), decreases the levels of 2-OG breaking the autoregulatory core NtcA-HetR. Early during the differentiation, PatS (or other derivative of it, see the text) diffuses along the filament inhibiting HetR. Both processes combined prevent the differentiation of the rest of vegetative cells and explain the formation of the pattern.

The up-regulatory loop of NtcA and HetR is essential for heterocyst differentiation [36–39]. However, the action of NtcA and HetR alone cannot explain pattern formation. Another transcription factor, PatS, inhibits the DNA-binding activity of HetR [8, 35, 40, 41]. This inhibitory behavior is essential for the communication with adjacent cells and thus to achieve the observed patterns of vegetative cells and heterocysts in cyanobacteria filaments (see Fig. 3). Furthermore, *patS* is strongly expressed in differentiating cells and mature heterocysts due to its upregulation by HetR [8]. A filament without *patS* develops multiple contiguous heterocysts (about a 30% of all cells as compared to the usual 10% in the wild-type filament). On the other hand, an over-expression of *patS* suppresses heterocyst differentiation [9]. Moreover, the addition to the growth medium of a synthetic peptide composed of the last five residues (RGSGR) of PatS (PatS5) inhibits heterocyst development, suggesting that PatS5 may be a diffusive mature form of PatS that stops the differentiation of the rest of vegetative cells of the filament [40]. A similar protein carrying the RGSGR pentapeptide, with a similar effect as that of PatS, is HetN [42]. A chain lacking both PatS and HetN leads to a lethal phenotype in which all cells differentiate [43].

The last stages of heterocyst development cause the physiological changes of the cell aimed at creating an anaerobic environment that sustains nitrogen fixation. To this end, two new membrane layers are biosynthesized to decrease the entry of oxygen into the cell [44]. The morphogenesis of these two layers is controlled by two family of genes, *hep* and *hgl*, that are indirectly up-regulated by HetR [35]. After these morphological changes the genes in charge of nitrogen fixation, *nif* genes, are expressed. These genes encode, among others, the enzyme nitrogenase, which ultimately performs nitrogen fixation.

The fixed nitrogen of the new heterocysts acts as an inhibitor of the differentiation together with PatS and HetN [45]. Thus, the diffusion of these inhibitors from heterocysts along the filament plays a key role in pattern maintenance (see Fig. 3). As a result of the differentiation heterocysts produce fixed nitrogen from N_2_ of the atmosphere and they interchange this nitrogen with the oxygen derivatives produced by the vegetative cells, in an illustration of the cooperative behavior between cell types in a multicellular organism.

### Regulatory equations

In this section we translate the genetic circuit previously described into a set of differential equations, for which we follow the derivation in [46–48]. Details are left to supplementary information (S1 text). To simplify notation, constants related to NtcA, HetR, PatS and cN are denoted with the letters *a, r, s*, and *n* respectively.

We start by looking at the transcription of *ntcA*, which is regulated by HetR and NtcA. We assume that the probability that NtcA binds the promoter in the absence of 2-OG can be neglected. Taking into account that both HetR and NtcA dimerize to bind DNA we find:

$$\begin{array}{c} v_{a}=L_{a}+\frac{v_{a}^{a}\kappa_{a}^{a}\left[\text{2-OG}\right]{\left[\text{NtcA}\right]}^{2}+v_{a}^{r}\kappa_{a}^{r}{\left[\text{HetR}\right]}^{2}+v_{a}^{ar}\kappa_{a}^{a}\kappa_{a}^{r}\left[\text{2-OG}\right]{\left[\text{NtcA}\right]}^{2}{\left[\text{HetR}\right]}^{2}}{\left(1+\kappa_{a}^{a}\left[\text{2-OG}\right]{\left[\text{NtcA}\right]}^{2}\right)\left(1+\kappa_{a}^{r}{\left[\text{HetR}\right]}^{2}\right)}, \end{array}$$ (1)

where *v*_*a*_ measures the production rate of NtcA in units of concentration per time, $v_{a}^{a}$, $v_{r}^{a}$ and $v_{ar}^{a}$ are the rates when only NtcA, only HetR or both are bound to DNA respectively, and $\kappa_{*}^{a}$ are the inverse of the effective dissociation constants of the compounds that bind DNA. *L*_*a*_, the so-called leak term, measures the basal production of *ntcA* in the absence of regulation. Subscripts and superscripts identify the binding site and the transcription factor for which the constants are given respectively.

Similarly we can obtain the transcription velocity for HetR. We assume that *hetR* is regulated by NtcA by means of a usual Hill function, yet the real process presents an intermediate, *nrrA*. To do so, we take into account that *nrrA* concentration relaxes rapidly to a limiting value. Furthermore, PatS affects the auto-regulatory loop of HetR. It has been suggested that PatS binds the binding site of HetR in the promoter of *hetR* preventing HetR binding [35]. These facts, along with the influence of 2-OG levels, provide with an expression for the transcription velocity:

$$\begin{array}{c} v_{r}=L_{r}+\frac{v_{r}^{a}\kappa_{r}^{a}\left[\text{2-OG}\right]{\left[\text{NtcA}\right]}^{2}\left(1+\kappa_{r}^{s}\left[\text{PatS}\right]\right)+v_{r}^{r}\kappa_{r}^{r}{\left[\text{HetR}\right]}^{2}+v_{r}^{ar}\kappa_{r}^{a}\kappa_{r}^{r}\left[\text{2-OG}\right]{\left[\text{NtcA}\right]}^{2}{\left[\text{HetR}\right]}^{2}}{\left(1+\kappa_{r}^{a}\left[\text{2-OG}\right]{\left[\text{NtcA}\right]}^{2}\right)\left(1+\kappa_{r}^{r}{\left[\text{HetR}\right]}^{2}+\kappa_{r}^{s}\left[\text{PatS}\right]\right)} \end{array}$$ (2)

HetR regulates most processes of the genetic circuit. It governs, among others, the transcription of *ntcA, patS, hep, hgl* and *nif* genes that lead to most of structural changes of the cell and to nitrogen fixation.

The inhibitor PatS is regulated by HetR, and we assume no other influence. For simplicity, we implicitly include the effect of HetN in PatS, as their action is expected to be equivalent (see the previous section). This gives the simple transcription velocity:

$$\begin{array}{c} v_{s}=L_{s}+\frac{v_{s}^{r}\kappa_{s}^{r}{\left[\text{HetR}\right]}^{2}}{1+\kappa_{s}^{r}{\left[\text{HetR}\right]}^{2}} \end{array}$$ (3)

Finally, we have to relate nitrogenase concentration [Ni] to that of combined Nitrogen [cN], both regulated by HetR and the levels of 2-OG [2-OG]. Let us begin by examining nitrogenase concentration, which is directly controlled by *nif* genes. Although this is not a direct process, we can assume, as we did for the NtcA-regulation of *hetR*, that *nif* genes are functionally governed by [HetR] following a typical Hill function. The nitrogenase production rate is given by:

$$\begin{array}{c} \frac{d\text{[Ni]}}{dt}=L_{Ni}+\frac{v_{Ni}^{r}\kappa_{Ni}^{r}{\text{[HetR]}}^{2}}{1+\kappa_{Ni}^{r}{\text{[HetR]}}^{2}}-\delta_{Ni}\text{[Ni]}. \end{array}$$ (4)

where *δ*_*Ni*_ represents the degradation rate of nitrogenase. We can effectively account for the lag introduced by intermediate processes not taken into account explicitly in the model by increasing the value of *δ*_Ni_ so that [Ni] relaxes more slowly. Assuming that nitrogenase produces fixed nitrogen at a constant rate, we arrive at the equation that governs cN levels in cyanobacteria:

$$\begin{array}{c} \frac{d\text{[cN]}}{dt}=L_{n}^{\prime }+v_{n}^{\prime }\text{[Ni]}-\delta_{n}^{\prime }\text{[cN]}, \end{array}$$ (5)

where $L_{n}^{\prime }$ represents the flux of cN from the exterior of the cell. Assuming that the levels of cN relax rapidly we solve Eq. (5) for the steady state. Substituting in Eq. (4) we find:

$$\begin{array}{c} \frac{d\text{[cN]}}{dt}=L_{n}+\frac{v_{n}^{r}\kappa_{n}^{r}{\text{[HetR]}}^{2}}{1+\kappa_{n}^{r}{\text{[HetR]}}^{2}}-\delta_{n}\text{[cN]}\;, \end{array}$$ (6)

where

$$\begin{array}{c} L_{n}=\frac{1}{\delta_{n}^{\prime }}\left(v_{n}^{\prime }L_{\text{Ni}}+\delta_{\text{Ni}}L_{n}^{\prime }\right),\;v_{n}^{r}=\frac{v_{n}^{\prime }}{\delta_{n}^{\prime }}v_{\text{Ni}}^{r},\;\delta_{n}=\delta_{\text{Ni}},\;\kappa_{n}^{r}=\kappa_{Ni}^{r}. \end{array}$$ (7)

To get a closed system of equations, we shall investigate the relation between cN and 2-OG. Both are related by means of the GS/GOGAT cycle (Fig. 2). Assuming the cycle is in equilibrium and reactions are grounded on the law of mass action, the following two conditions must be satisfied:

$$\begin{array}{c} \left[\text{glutamate}\right]=\kappa_{\leftarrow }\left[\text{glutamine}\right]\left[\text{2-OG}\right],\;\left[\text{glutamine}\right]=\kappa_{\to }\left[\text{glutamate}\right]\left[\text{cN}\right], \end{array}$$ (8)

which lead to the relation:

$$\begin{array}{c} \text{[2-OG]}=\frac{1}{\kappa_{\leftarrow }\kappa_{\to }\text{[cN]}}. \end{array}$$ (9)

However, this expression does not behave properly for small concentrations of cN, which are expected under cN deprivation: 2-OG levels would increase without limit. In fact, 2-OG production is controlled by some processes that are not considered in this work and so its value must be limited. We can effectively include such a limiting value by means of a translation on [cN] in Eq. (9)

$$\begin{array}{c} \text{[2-OG]}=\frac{1}{\kappa_{\text{2-OG}}+\kappa_{\leftarrow }\kappa_{\to }\text{[cN]}}\;, \end{array}$$ (10)

which reaches the maximum value [2-OG]_max_ = 1/*κ*_2-OG_ at [cN] = 0.

Finally we introduce the differential equations governing cyanobacterial reaction to nitrogen deprivation. They represent the temporal variation of the most important factors of the genetic circuit, namely NtcA, HetR, PatS and cN. Using the production rates (1), (2), (3), (6) and introducing degradation rates constants, δ_*_, we find:

$$\begin{array}{l} \frac{dq_{a}}{d\tau }=l_{a}+\frac{\beta_{a}^{a}\gamma_{a}^{a}q_{a}^{2}+\beta_{a}^{r}\gamma_{a}^{r}q_{r}^{2}(1+q_{n})+\beta_{a}^{ar}\gamma_{a}^{a}q_{a}^{2}\gamma_{a}^{r}q_{r}^{2}}{\left(1+q_{n}+\gamma_{a}^{a}q_{a}^{2})(1+\gamma_{a}^{r}q_{r}^{2}\right)}-d_{a}q_{a},\\ \frac{dq_{r}}{d\tau }=l_{r}+\frac{\beta_{r}^{a}q_{a}^{2}(1+q_{s})+\beta_{r}^{r}q_{r}^{2}(1+q_{n})+\beta_{r}^{ar}q_{a}^{2}q_{r}^{2}}{\left(1+q_{n}+q_{a}^{2})(1+q_{s}+q_{r}^{2}\right)}-q_{r},\\ \frac{dq_{s}}{d\tau }=l_{s}+\frac{\beta_{s}^{r}\gamma_{s}^{r}q_{r}^{2}}{1+\gamma_{s}^{r}q_{r}^{2}}-d_{s}q_{s},\\ \frac{dq_{n}}{d\tau }=l_{n}+\frac{\beta_{n}^{r}\gamma_{n}^{r}q_{r}^{2}}{1+\gamma_{n}^{r}q_{r}^{2}}-d_{n}q_{n}, \end{array}$$ (11)

where we have introduced the dimensionless variables:

$$q_{a}=\underset{\phi_{a}}{\underset{︸}{\sqrt{\frac{\kappa_{r}^{a}}{\kappa_{\text{2-OG}}}}}}[\text{NtcA}],\;q_{r}=\underset{\phi_{r}}{\underset{︸}{\sqrt{\kappa_{r}^{r}}}}[\text{HetR}],\;q_{s}=\underset{\phi_{s}}{\underset{︸}{\kappa_{r}^{s}}}[\text{PatS}],\;q_{n}=\underset{\phi_{n}}{\underset{︸}{\frac{\kappa_{\leftarrow }\kappa_{\to }}{\kappa_{\text{2-OG}}}}}[\text{cN}],\;\tau =\delta_{r}t,$$ (12)

and the constants

$$\begin{array}{c} l_{*}=\frac{L_{*}\phi_{*}}{\delta_{r}},\;\beta_{*}^{•}=\frac{v_{*}^{•}\phi_{*}}{\delta_{r}},\;\gamma_{*}^{•}=\frac{\kappa_{*}^{•}}{\kappa_{r}^{•}},\;d_{*}=\frac{\delta_{*}}{\delta_{r}}. \end{array}$$ (13)

Let us finally stress that this is a *deterministic* model for a single cyanobacterium. The study of the cyanobacterial filament is left to the final section. We show that the main modification will be adding diffusion processes for the inhibitors PatS and cN through the chain. An important ingredient in pattern formation, noise, will be also added to the equations.

### Unicellular dynamics

In this section we analyze the dynamical system (11) for a set of constants (Table 1) that exhibit both the dynamical and the structural properties of heterocyst differentiation. Following the usual practice in the analysis of dynamical systems, we study the basic properties of equations (11), such as fixed points and linear stability analysis, to analyze the key features leading to heterocyst differentiation.

**Table 1:** Parameters for Eq. (11) that reproduce heterocyst formation under noisy conditions and pattern formation when PatS and cN diffuse along a filament of cyanobacteria.

Constants			
*l*_*a*_ = 0.2	*l*_*r*_ = 0.01	*l*_*s*_ = 0.0001	*l*_*n*_ = 0
*d*_*a*_ = 0.7	*d*_*s*_ = 0.05	*d*_*n*_ = 0.01	$\beta_{a}^{a}=4$
$\beta_{a}^{r}=4$	$\beta_{a}^{ar}=8$	$\beta_{r}^{a}=1$	$\beta_{r}^{r}=1$
$\beta_{a}^{ar}=3$	$\beta_{s}^{r}=0.385$	$\beta_{n}^{r}=0.06$	$\gamma_{a}^{a}=3$
$\gamma_{a}^{r}=2.4$	$\gamma_{s}^{r}=1.2$	$\gamma_{n}^{r}=2.75$	

Taking into account the difference between the relaxation times of the constituents of the model, given by the inverses of d_*_ (see Table 1), we can interpret it as composed of two temporally separated systems: a rapid one, formed by HetR and NtcA, showing fast dynamics that relaxes to its steady state almost instantaneously and a slow one, composed of PatS and cN, whose evolution is dictated by the values of HetR and NctA in their instantaneous equilibrium. This corresponds to an adiabatic elimination technique [49] that helps in reducing the complexity of the dynamical system by splitting it into two simpler interdependent subsystems.

First, we look at the fixed points of *q*_*a*_ and *q*_*r*_ for each pair of values of *q*_*s*_ and *q*_*n*_

$$\begin{array}{c} f_{a}\left(q_{s},q_{n}\right)=\frac{dq_{a}}{d\tau }=0,\;f_{r}\left(q_{s},q_{n}\right)=\frac{dq_{r}}{d\tau }=0. \end{array}$$ (14)

**Fig 4:**
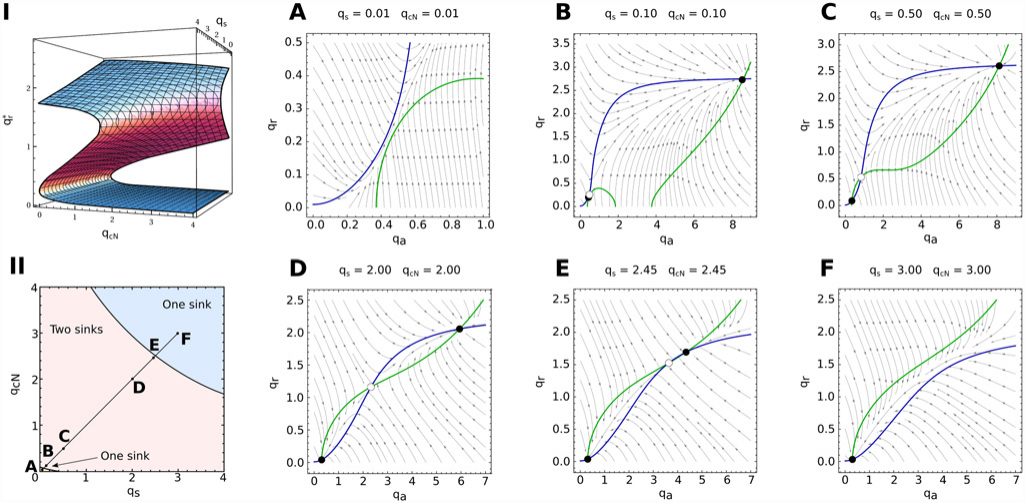
Adiabatic elimination of the fast variables *q*_*r*_ and *q*_*a*_. Due to the fast dynamics that HetR and NtcA exhibit, we can approach the treatment of the system by adopting a point of view that follows the slower variables *q*_*s*_ and *q*_*n*_. From this viewpoint, the time-evolution of the pair (*q*_*s*_(*t*), *q*_*n*_(*t*)) is considered by assuming that *q*_*r*_ and *q*_*a*_ instantaneously relax to an equilibrium, which corresponds to a sink ($q_{r}^{*}$, $q_{a}^{*}$) for the fixed pair (*q*_*s*_(*t*), *q*_*n*_(*t*)). Depending on the region of the (*q*_*s*_, *q*_*n*_)-plane, there are three fixed points (two sinks corresponding to the highest and the lowest concentrations respectively and a saddle in the middle) or one (a sink) for *q*_*r*_ and *q*_*a*_ (I and II). There are two one-sink regions that are separated from the two-sink region by saddle-node bifurcations (A-F). Sinks and saddles are represented by filled and unfilled circles respectively and arrows indicate the flow of the dynamics. We can then imagine the dynamics of *q*_*s*_ and *q*_*n*_ as evolving either in the bottom or in the top branch of I. In the two-sink region, both branches are plausible and the history of the dynamics determine the solution (hysteresis effect): a dynamics in a branch will continue in it until experiencing a bifurcation in the (*q*_*r*_, *q*_*a*_) plane (see Fig. 5 for examples).

The numerical solution to this problem is sketched in Fig. 4. We find three different branches of solutions that coexist in some regions. The fixed points on the lower and upper branches are always stable (blue region in Fig. 4I) and those lying on the middle branch (red region) are saddles. Transitions between the regions with one and three fixed points correspond to saddle-node bifurcations in which the middle branch of solutions coalesce with the lower and the upper one respectively. The basins of attraction of both stable fixed points are separated by the stable manifold of the saddle point (Fig. 4II).

In the bistable region the system behaves as a switch that can be either OFF in a vegetative state (lower branch, with a small production of HetR and NtcA) or ON in a heterocyst state (upper branch, with a high production of HetR and NtcA). A sufficient large perturbation may result in the system crossing the manifold of the saddle and falling into the other stable branch of solutions. The distance between the saddle and the nodes determines the size of the perturbation needed to activate or inactivate the system.

**Fig 5:**
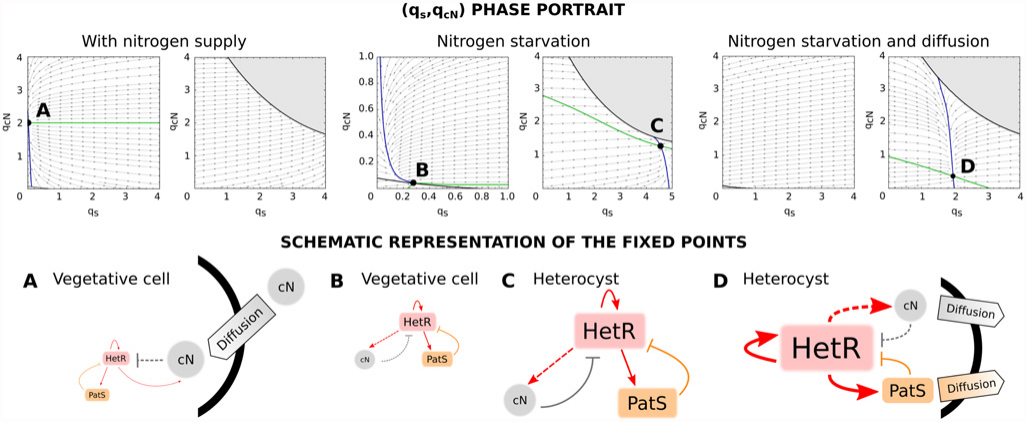
States of a cyanobacterium when subjected to different conditions of nitrogen and diffusion. When the cell is provided of cN (*l*_*n*_ = 0.03), there is only one stable fixed point (A) in the bottom branch, which corresponds to a state in which the production of both HetR and PatS is minimum (vegetative state). When subjected to nitrogen deprivation (*l*_*n*_ = 0), there are two stable fixed points (B and C) each one in a different branch. The first point (B) is a vegetative state in which there exists an equilibrium between a small production of HetR, PatS and cN. The same kind of equilibrium is present in the second fixed point (C) but in this case the production of all TFs and cN is high (heterocyst steady state). When the cell is exposed to nitrogen stress its trajectory evolves from A to the steady state B and thus it remains vegetative. Assuming some diffusion of cN and PatS from the cell (*l*_*s*_ = −0.2 and *l*_*n*_ = −0.002), the only stable state (D) corresponds to a heterocyst state with high levels of production of HetR, cN and PatS, being the latter transported to the surroundings of the cell.

With (14) solved, we can apply the solution to calculate the effective field sensed by the (*q*_*s*_, *q*_*n*_) pair. In the regions showing bistability the field takes two very different forms, one corresponding to the values of the lower branch and another corresponding to those of the upper one (Fig. 5). We expect a *hysteresis effect*: if initially the dynamics lies on a particular branch it will remain on it unless a fluctuation or a bifurcation makes the system jump to the other branch.

In the presence of cN (Fig. 5A) we find only one stable fixed point that corresponds to a vegetative state (lower branch). The upper branch is completely unstable: any dynamics lying on it will fall down to the lower branch and eventually be attracted to the vegetative sink. The steady state is very robust against perturbations since it is far from the bifurcation region and there is a significant distance to the saddle in the *q*_*r*_ − *q*_*a*_ plane.

By reducing the flow of cN from the exterior of the cell (*l*_*n*_ = 0) we find that a stable fixed point appears in the upper branch, a heterocyst state, while the vegetative state gets closer to the bifurcation region, thus becoming more susceptible to perturbations that can make the system reach the upper branch. In the absence of cN, the cyanobacterium would evolve from state A to state B in the lower branch until a perturbation pushes it to the upper branch, eventually becoming an heterocyst due to the field acting on that branch (Fig. 5B and C).

Diffusion protects cells in the neighborhood of the newly formed heterocyst to initiate the differentiation: as heterocysts are producers of cN and PatS, the vegetative fixed point of the cells in its neighborhood will move towards an A-like state, thus becoming more stable to perturbations. The heterocyst fixed point also becomes more stable due to diffusion, since its production of inhibitors is distributed among other cells (see Fig. 5D).

### Strains of cyanobacteria. Heterocyst patterns

In the previous section, we introduced a single cell model for the cyanobacteria reaction to nitrogen-limiting conditions. There we have shown that, for a specific range of parameters, the model exhibits features that would lead to heterocyst development under noisy conditions. Nevertheless, the model should be extended to cyanobacteria chains to account for heterocyst development since, as previously noted, isolated cyanobacteria do not become heterocysts by themselves; the action of the chain is needed to generate heterocysts.

In this section, we extend the previous results and consider a chain of vegetative cells facing nitrogen deprivation. The main modification is the introduction of diffusion of PatS and cN along the cyanobacteria chain. For this purpose we add to Eq. (12) the discrete version of the diffusion equation:

$$\begin{array}{c} \frac{dC_{i}}{dt}=D_{C}\left(C_{i+1}+C_{i-1}-2C_{i}\right). \end{array}$$ (15)

where *D*_*C*_ is called the *diffusion constant* of the element C. Now, it is straightforward to introduce PatS and cN diffusion into the equations. The dynamics of cell *i* is characterized by the following set of equations:

$$\begin{array}{l} \frac{dq_{i,a}}{d\tau }=l_{a}+\frac{\beta_{a}^{a}\gamma_{a}^{a}q_{a}^{2}+\beta_{a}^{r}\gamma_{a}^{r}q_{r}^{2}(1+q_{n})+\beta_{a}^{ar}\gamma_{a}^{a}q_{a}^{2}\gamma_{a}^{r}q_{r}^{2}}{\left(1+q_{n}+\gamma_{a}^{a}q_{a}^{2})(1+\gamma_{a}^{r}q_{r}^{2}\right)}-d_{a}q_{i,a}+G_{i,a}(t),\\ \frac{dq_{i,r}}{d\tau }=l_{r}+\frac{\beta_{r}^{a}q_{a}^{2}(1+q_{s})+\beta_{r}^{r}q_{r}^{2}(1+q_{n})+\beta_{r}^{ar}q_{a}^{2}q_{r}^{2}}{\left(1+q_{n}+q_{a}^{2})(1+q_{s}+q_{r}^{2}\right)}-q_{i,r}+G_{i,r}(t),\\ \frac{dq_{i,s}}{d\tau }=l_{s}+\frac{\beta_{s}^{r}\gamma_{s}^{r}q_{r}^{2}}{1+K_{s}^{r}q_{r}^{2}}-d_{s}q_{i,s}+D_{s}\left(q_{i+1,s}+q_{i-1,s}-2q_{i,s}\right)+G_{i,s}(t),\\ \frac{dq_{i,n}}{d\tau }=l_{n}+\frac{\beta_{n}^{r}\gamma_{n}^{r}q_{r}^{2}}{1+\gamma_{n}^{r}q_{r}^{2}}-d_{n}q_{i,n}+D_{n}\left(q_{i+1,n}+q_{i-1,n}-2q_{i,n}\right)+G_{i,n}(t), \end{array}$$ (16)

which constitutes the model for a cyanobacteria filament. To account for environment variability we add white noise, *G*_*i*, *_(*t*), of the same amplitude, ⟨*G*_*i*, *_(*t*)*G*_*i*, *_(*t* ^′^)⟩ = *ξδ*(*t* − *t* ^′^), for all the components of the system. Based on these equations, we investigate the conditions that lead to a heterocyst pattern. It is easy to notice that they correspond to an activator-inhibitor system of cells coupled in a reaction-diffusion scheme [50]. This kind of system produces regular pattern formation [51–53]. Turing (linear stability) analysis of equations (16) (see S2 text) provides insight on the periodicity of patterns. It is interesting to show that the minimum periodicity observed in such analysis is larger than 1, which means that a single bacteria is unable to differentiate.

We performed the direct integration of equations (16) for chains of 200 cyanobacteria. We used a Runge-Kutta method for the numerical integration of stochastic differential equations (see Methods) [54]. All simulations were performed with periodic boundary conditions, i.e. emulating a circular filament, for simplicity. We have also tested the more realistic no flux boundary conditions and find no change in gene dynamics and heterocyst patterns in the interior of the filament. This shows that the effect of boundary conditions is highly localized around the borders. In simulations with no flux boundary conditions no heterocysts were found in the border, which is in good agreement with experimental observations [55]. The level of noise that best reproduces heterocyst pattern is *ξ* = 0.001 for the set of parameters of Table 1. Importantly, isolated cells do not initiate differentiation with this level of noise, in agreement with the results from the linear stability analysis. Diffusion constants have been set to *D*_*s*_ = 0.1 and *D*_*n*_ = 0.2. Heterocysts patterns develop for different levels of noise and diffusion constants, but the model parameters, which characterize cell response to nitrogen deprivation, should change accordingly. This correlation between noise, diffusion and model parameters supports the idea that cyanobacteria have evolved towards a better response to the normal levels of noise in their environment.

**Fig 6:**
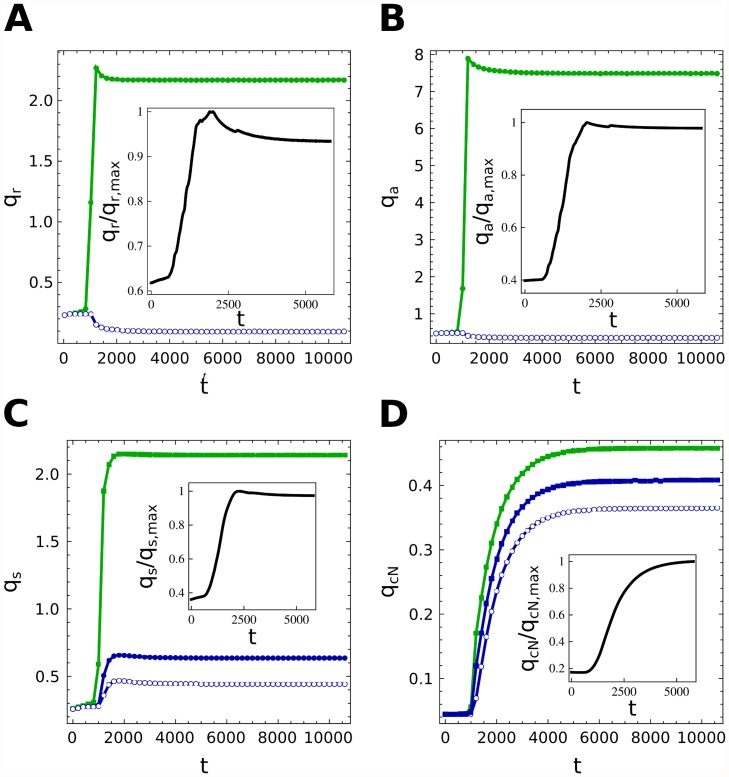
Time evolution of molecular concentrations. Time evolution of the main components of the differentiation in heterocysts (green) and vegetative cells (blue). Averages along the filament are also presented (black). Heterocysts, due to the early diffusion, evolve toward steady states of the type D of Fig. 5 characterized by high levels of HetR and NtcA while vegetative cells present very low concentrations of them (A and B). The levels of PatS and cN in vegetative cells depend on their distance to close heterocysts: C and D show the concentrations of PatS and cN in a heterocyst and in its first two neighbouring vegetative cells, which clearly highlight the effect of diffusion along the filament.

In Fig. 6 we show the dynamics that the 4 variables exhibit when the filament is deprived of cN. We observe that the filament concentrations relax to the constant protein levels of the vegetative state we showed in the previous chapter. Then, due to the coupled action of noise and diffusion, some cells start to differentiate. As new forming heterocysts appear, their production and exportation of inhibitors to the surrounding cells make the latter more stable to perturbations stopping their differentiation. The model reproduces very well the initial peak that both NtcA and HetR present experimentally [35, 56]. PatS increases more slowly to its steady value reducing the levels of NtcA and HetR and, finally, cN is generated by heterocysts stabilizing the pattern.

**Fig 7:**
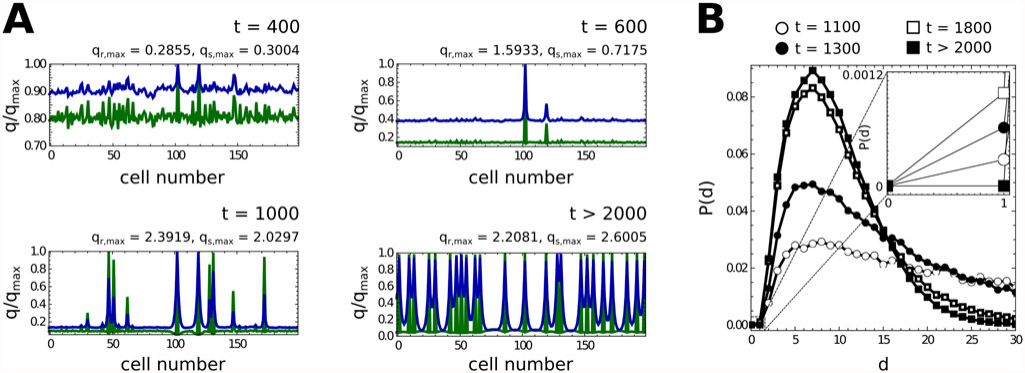
Heterocyst pattern. Time-evolution of the pattern of heterocysts (A) and of the probability distribution of the distance between consecutive heterocysts (B). Green and blue curves represent the concentration profiles of HetR and PatS (NtcA and cN are not presented since their behavior along the filament is comparable to that of HetR and PatS, see Fig. 6 to see the similarities). Small perturbations along the filament of vegetative cells (initially in the steady state B of Fig. 5) are amplified due to diffusion processes in a demonstration of Turing’s theory [51]. New heterocysts appear in regions that are not dominated by the action of other heterocysts. Finally, the competition between nearby differentiating cells ceases the differentiation of some of them, as observed in B: consecutive heterocysts, which are created by strong perturbations, finally disapear due to the aforementioned competition. The final pattern presents localized levels of HetR (heterocysts) and a diffusive-like behavior of PatS, as expected.

Fig. 7 shows the evolution of the profile for a 200 cells chain of cyanobacteria. We observe that heterocysts progressively appear in those regions in which other heterocysts do not have effect (*i.e.* those vegetative cells that are not supplied of sufficient cN and PatS). Finally, a semiregular pattern is generated. PatS and cN diffuse along the filament exhibiting smooth variations between vegetative cells and heterocysts, while HetR and NtcA present very abrupt variations between cell types.

Finally, in Fig. 7B we show the time-evolution of the histogram for the distance between two consecutive heterocysts. It should be stressed that although initially some close heterocysts appear, they are eliminated by the non-linear action of the system during the differentiation process. Close heterocysts compete for the same region of action (the same vegetative cells that consume their PatS and cN) and then they cannot reach the optimal heterocyst state, which is stable to slight perturbations. Finally, one of them falls down from the upper branch becoming a vegetative cell. This behavior is typically observed experimentally [4, 9]. The final histogram can be nicely fitted by a Γ-distribution, implying Poisson-distributed wait times for the underlying noise driven process.

## Discussion

The study of cell differentiation and its underlying mechanisms constitutes one of most intriguing problems in biology. This phenomenon is the basis for multicellular organisms and pattern formation. The approach presented here deals with a simple system, heterocyst formation in cyanobacteria filaments, yet complex enough to capture the main ingredients of some of the mechanisms for cell differentiation and pattern formation under external driving. The knowledge of the basic regulatory genes and their corresponding interactions allows for a detailed description of cell dynamics. We have derived the evolution equations of the involved genes based on the statistical mechanics of their corresponding regulatory processes. This allows us to obtain a detailed description of the *continuous time dynamics* of the main regulatory proteins, in contrast to other discrete approximations based in boolean dynamics [15]. This kind of analysis has been shown to describe successfully other time dependent phenomena concerning cyanobacteria such as their circadian cycles [57]. The analysis of the unicellular dynamics has revealed that the two cellular stable states, vegetative and heterocyst cells, appear as attractors of the non-linear dynamics of the regulatory equations. However, the study of many coupled cells is needed as cyanobacteria do not differentiate when isolated.

The model is rounded off by coupling a number of cells in a one-dimensional array so that combined nitrogen and PatS can diffuse along the cellular chain. We have shown that one important ingredient affecting the dynamical behavior of the chain is noise, which plays a key role in onset of the pattern formation, *i*.e., the transition from the initial chain of vegetative cells to the steady state in which heterocysts coexist with vegetative cyanobacteria. Thus, the appearance of differentiation is, in our model, a pure stochastic event. The cooperative character of the filament is clear from the amount of noise needed to start the differentiation process which appears significantly smaller than that needed in isolated cells. The source of noise as well as its biological consequences is, nowadays a current topic of research [58]. In fact, at its initial state, differentiation of cells appears randomly along the filament, but shortly after its onset a characteristic distribution of heterocyst emerges. This distribution can be compared with the experimental one with a fairly good agreement [41].

Although the model presented here integrates both the internal cell dynamics and the coupling between cells via diffusion, there exist other ingredients that can be also incorporated. One issue that have not been considered in this work is the replication of vegetative cells. This effect has been taken into account in [14]. Although this improvement is relevant, it only affects, in our approach, to the mean separation between heterocysts, by opening a gap in the Γ-function shape of Fig. 7B and thus approaching better to the experimental distribution.

Other improvements to the approach presented here will come from the availability of more experimental data. Unlike other approaches [13] in which comparison is done (globally) with heterocyst distributions, our work would allow for a qualitative comparison of each component involved in the differentiation (see Fig. 6). Unfortunately, there is not enough experimental data to make a detailed fit so to extract reliable parameters. The availability of such data is extremely important both for having a better set of model parameters and to validate new models. A complete understanding of the mechanism that derive in phenotypic differentiation is the first step for a modular comprehension of the whole cell [59].

## Methods

To reproduce the dynamics of Eq. (16) we make use of the integration scheme proposed in [54]. Eq. (16) is a set of stochastic differential equations (SDE) so its numerical integration requires generating a statistical representative trajectory for a discrete set of time-values. A SDE of the form

$$\begin{array}{c} \dot{x}=f\left(x\right)+G\left(t\right), \end{array}$$ (17)

where *G*(*t*) is a Gaussian white noise with

$$\begin{array}{c} \left\langle G\left(t\right)\right\rangle =0,\;\text{and,}\;\left\langle G\left(t\right)G\left(t^{\prime }\right)\right\rangle =\xi \delta \left(t^{\prime }-t\right), \end{array}$$

can be integrated through a Runge-Kutta integration algorithm by adding a particular Gaussian signal at each stage of the scheme. This algorithm coincides with the usual Runge-Kutta scheme for *ξ* = 0. In this work we have employed a 3_*O*_4_*S*_2_*G*_ algorithm, which is correct up to 3^*th*^ order, is developed in 4 stages and uses 2 independent Gaussian random variables.

## Supporting Information

S1 Text Regulatory equations: a statistical mechanics approach. (PDF)

S2 Text Turing linear stability analysis. (PDF)
