# Supplementary material for: An Integrative Approach for Modeling and Simulation of Heterocyst Pattern Formation in Cyanobacteria Filaments
Source: PLoS Comput Biol. 2015 Mar 27;11(3):e1004129. doi: 10.1371/journal.pcbi.1004129 (PMC4376521; doi:10.1371/journal.pcbi.1004129)
Supplement: S1 Text — (PDF) [file pcbi.1004129.s001.pdf]

Text S1: Regulatory equations: a statistical mechanics approach

Alejandro Torres-Sánchez[1,2], Jesus Gómez-Gardeñes[1,3], Fernando Falo[1,3]
[1]Departamento de Física de la Materia Condensada, Universidad de Zaragoza, Zaragoza, Spain.
[2] Laboratori de Càlcul Numèric, Universitat Politècnica de Catalunya-Barcelonatech, Barcelona, Spain.
[3]Instituto de Biocomputación y Física de Sistemas Complejos (BIFI), Universidad de Zaragoza, Zaragoza, Spain.

To derive the equations regulating transcription processes during heterocyst differentiation, we follow an approach similar to the detailed in [46, 47, 48]. This is a thermodynamic approach to transcription in which binding sites are considered two-states systems, either empty or containing a binding protein. The probability that a given transcription factor (TF) is bound to its binding site is given by the Arrhenius formula

$$p_{\mathrm{TF}} = \frac{[\mathrm{TF}]K_{\mathrm{TF}}}{1+[\mathrm{TF}]K_{\mathrm{TF}}} = \frac{q_{\mathrm{TF}}}{1+q_{\mathrm{TF}}} \tag{1}$$

where [TF] is the concentration of the TF, K_{TF} is the inverse of the effective dissociation constant, which represents the concentration of half-maximal occupation, and $q_{\mathrm{TF}} = [\mathrm{TF}]K_{\mathrm{TF}}$ is called the binding affinity. The denominator of Eq. (1) is nothing but the canonical partition function of the promoter $Z = 1 + [\mathrm{TF}]K_{\mathrm{TF}}$, representing the Boltzmann-weighted sum of possible states of the binding site. Transcription starts with the binding of RNAp, which in the absence of interactions with TFs follows the probability law of Eq. (1):

$$p_{\mathrm{RNAp}} = \frac{q_{\mathrm{RNAp}}}{1+q_{\mathrm{RNAp}}} \tag{2}$$

Let us examine the case in which RNAp interacts with a TF within the promoter. In this case the partition function is

$$Z_{\mathrm{RNAp,TF}} = 1 + [\mathrm{TF}]K_{\mathrm{TF}} + [\mathrm{RNAp}]K_{\mathrm{RNAp}} + [\mathrm{RNAp}][\mathrm{TF}]K_{\mathrm{RNAp,TF}} \tag{3}$$

with $K_{\mathrm{RNAp,TF}}$ the inverse dissociation constant of the complex RNAp&TF that can be higher than $K_{\mathrm{TF}}K_{\mathrm{RNAp}}$ if the interaction of the two proteins within the promoter is attractive or smaller if the interaction is repulsive. In the first case we say that the TF is an inhibitor while in the second case we say that the TF is an activator. We are interested in the probability that RNAp is bound to its binding site. This probability is

$$\begin{aligned} p_{\mathrm{RNAp}} &= \frac{[\mathrm{RNAp}]K_{\mathrm{RNAp}} + [\mathrm{RNAp}][\mathrm{TF}]K_{\mathrm{RNAp,TF}}}{1 + [\mathrm{TF}]K_{\mathrm{TF}} + [\mathrm{RNAp}]K_{\mathrm{RNAp}} + [\mathrm{RNAp}][\mathrm{TF}]K_{\mathrm{RNAp,TF}}} = \\ &= \frac{q_{\mathrm{RNAp}}\left(1+q_{\mathrm{TF}}\omega_{\mathrm{RNAp,TF}}\right)}{1+q_{\mathrm{TF}}+q_{\mathrm{RNAp}}\left(1+q_{\mathrm{TF}}\omega_{\mathrm{RNAp,TF}}\right)}, \end{aligned} \tag{4}$$

where we have used the definitions $q_{\rm TF} = [{\rm TF}]K_{\rm TF}$, $q_{\rm RNAp} = [{\rm RNAp}]K_{\rm RNAp}$ and $\omega_{\rm RNAp,TF} = K_{\rm RNAp,TF}/(K_{\rm TF}K_{\rm RNAp})$. Now, assuming that [RNAp] does not vary during the transcription process (or that it is not the limiting factor of transcription) and that transcription (which we remind is nothing but the production of mRNA) takes place at a given velocity ν when RNAp is bound to the promoter, we can find the relation between $q_{\rm TF}$ and the transcription velocity $v_{\rm mRNA}$:

$$v_{\rm mRNA} = L^{\rm A} + v_{\rm TF}\frac{q_{\rm TF}\kappa^{\rm A}_{\rm TF}}{1+q_{\rm TF}\kappa^{\rm A}_{\rm TF}}, \tag{5}$$

for the case in which the TF is an activator ($\omega_{\rm RNAp,TF} > 1$) or

$$v_{\rm mRNA} = L^{\rm I} + v_{\rm TF}\frac{1}{1+q_{\rm TF}\kappa^{\rm I}}, \tag{6}$$

in the case of an inhibitor ($\omega_{\rm RNAp,TF} < 1$). The remaining constants are

$$\begin{aligned} v_{\rm TF} &= \nu\frac{q_{\rm RNAp}\,|\omega_{\rm RNAp,TF}-1|}{(1+q_{\rm RNAp})(1+q_{\rm RNAp}\omega_{\rm RNAp,TF})}, \\ \kappa^{\rm A} &= \frac{1}{\kappa^{\rm I}} = \frac{1+\omega_{\rm RNAp,TF}q_{\rm RNAp}}{1+q_{\rm RNAp}}, \\ L^{\rm A}_{\rm mRNA} &= \nu\frac{q_{\rm RNAp}}{1+q_{\rm RNAp}}, \\ L^{\rm I}_{\rm mRNA} &= \nu\frac{q_{\rm RNAp}\omega_{\rm RNAp,TF}}{1+q_{\rm RNAp}\omega_{\rm RNAp,TF}}. \end{aligned} \tag{7}$$

These equations are of the form of the Michaelis-Menten equations of reaction kinetics with a leak term, represented by L^A or L^I depending on the case, that stands for the production of mRNA in the absence of regulation. This example shows the main features of the statistical mechanics approach to transcription. More complex transcription processes can be dealt with in a similar way by computing their corresponding partition function and counting the RNAp-active states.

For all processes in the article we assume that TFs do not interact within the promoter but rather that they cooperatively affect the velocity at which transcription takes place. This is the simplest way in which we can consider the interaction between different TFs and, on the other hand, it is rich enough to represent the main features of the transcription processes we need to account for.

Let us sketch, for instance, the regulation of *ntcA* in heterocyst development (see main text). *ntcA* is regulated partly by NtcA (in its dimer configuration) and 2-OG and also by HetR. The partition function in this case is:

$$\begin{aligned} Z_{\rm RNAp,NtcA\&2\text{-}OG,HetR} = 1 &+ [{\rm RNAp}]K_{\rm RNAp} + [{\rm NtcA}]^2[\text{2-OG}]K_{\rm NtcA2,2\text{-}OG} + \\ &+ [{\rm RNAp}][{\rm NtcA}]^2[\text{2-OG}]K_{\rm RNAp,NtcA2,2\text{-}OG} \\ &+ [{\rm HetR}]^2K_{\rm HetR2} + [{\rm RNAp}][{\rm HetR}]^2K_{\rm RNAp,HetR2} + \\ &+ [{\rm HetR}]^2[{\rm NtcA}]^2[\text{2-OG}]K_{\rm NtcA2,2\text{-}OG}K_{\rm HetR2} \\ &+ [{\rm RNAp}][{\rm HetR}]^2[{\rm NtcA}]^2[\text{2-OG}]K_{\rm RNAp,NtcA2,2\text{-}OG}K_{\rm RNAp,HetR2} \end{aligned} \tag{8}$$

Both NtcA and HetR acts as activators, and we finally arrive to the transcription velocity

$$v_a = L_a + \frac{v^a_a\kappa^a_a[\text{2-OG}][{\rm NtcA}]^2 + v^r_a\kappa^r_a[{\rm HetR}]^2 + v^{ar}_a\kappa^a_a\kappa^r_a[\text{2-OG}][{\rm NtcA}]^2[{\rm HetR}]^2}{(1+\kappa^a_a[\text{2-OG}][{\rm NtcA}]^2)(1+\kappa^r_a[{\rm HetR}]^2)} \tag{9}$$

where v_a^a, v_a^r and v_a^{ar} represent the effective transcription velocity when NtcA, HetR or both are bound to DNA respectively. The constants κ are obtained from K by eliminating q_{RNAp} from the equations following a procedure similar to that of Eq. (5) and can be thought as the inverse of effective dissociation constants associated with the binding of the different compounds.

Finally, we consider translation (the process by which mRNA is transformed into the corresponding protein) is produced at a constant rate η per mole of mRNA. Therefore, the concentration of a given TF is given by:

$$\frac{d[\mathrm{TF}]}{dt} = \eta_{\mathrm{TF}}[\mathrm{mRNA}_{\mathrm{TF}}] - \delta_{\mathrm{TF}}[\mathrm{TF}], \tag{10}$$

with δ_{TF} the inverse of the mean lifetime of the TF. On the other hand, the dynamics for concentration of mRNA is

$$\frac{d[\mathrm{mRNA}_{\mathrm{TF}}]}{dt} = v_{\mathrm{mRNA}_{\mathrm{TF}}} - \delta_{\mathrm{mRNA}_{\mathrm{TF}}}[\mathrm{mRNA}_{\mathrm{TF}}], \tag{11}$$

with $\delta_{\mathrm{mRNA}_{\mathrm{TF}}}$ the inverse of the mean lifetime of the mRNA. Usually, $\delta_{\mathrm{mRNA}_{\mathrm{TF}}} >> \delta_{\mathrm{TF}}$ and, as we are interested in [TF], we can assume that from the viewpoint of the characteristic dynamics of the TF, [mRNA] relaxes instantaneously to its equilibrium value:

$$[\mathrm{mRNA}_{\mathrm{TF}}] = \frac{v_{\mathrm{mRNA}_{\mathrm{TF}}}}{\delta_{\mathrm{mRNA}_{\mathrm{TF}}}}, \tag{12}$$

so

$$\frac{d[\mathrm{TF}]}{dt} = \eta \frac{v_{\mathrm{mRNA}_{\mathrm{TF}}}}{\delta_{\mathrm{mRNA}_{\mathrm{TF}}}} - \delta_{\mathrm{TF}}[\mathrm{TF}]. \tag{13}$$

In an abuse of notation, we redefine the variables $v_*^\bullet$ and l_* as $v_*^\bullet \eta_*/\delta_{\mathrm{mRNA}_*}$ and $v_*^\bullet \eta_*/\delta_{\mathrm{mRNA}_*}$ respectively to express the effective constants in Eq. (13). For instance, we find

$$\frac{d[\mathrm{NtcA}]}{dt} = L_a + \frac{v_a^a \kappa_a^a [\text{2-OG}][\mathrm{NtcA}]^2 + v_a^r \kappa_a^r [\mathrm{HetR}]^2 + v_a^{ar} \kappa_a^a \kappa_a^r [\text{2-OG}][\mathrm{NtcA}]^2 [\mathrm{HetR}]^2}{(1 + \kappa_a^a [\text{2-OG}][\mathrm{NtcA}]^2)(1 + \kappa_a^r [\mathrm{HetR}]^2)} - \delta_a [\mathrm{NtcA}] \tag{14}$$
