# Supplementary material for: An Integrative Approach for Modeling and Simulation of Heterocyst Pattern Formation in Cyanobacteria Filaments
Source: PLoS Comput Biol. 2015 Mar 27;11(3):e1004129. doi: 10.1371/journal.pcbi.1004129 (PMC4376521; doi:10.1371/journal.pcbi.1004129)
Supplement: S2 Text — (PDF) [file pcbi.1004129.s002.pdf]

Text S2: Turing linear stability analysis

Alejandro Torres-Sánchez[1,2], Jesus Gómez-Gardeñes[1,3], Fernando Falo[1,3]
[1]Departamento de Física de la Materia Condensada, Universidad de Zaragoza, Zaragoza, Spain.
[2] Laboratori de Càlcul Numèric, Universitat Politècnica de Catalunya-Barcelonatech, Barcelona, Spain.
[3]Instituto de Biocomputación y Física de Sistemas Complejos (BIFI), Universidad de Zaragoza, Zaragoza, Spain.

Here we discuss the Turing stability analysis of our system [51, 53]. Let us first discuss the general theory. We consider a chain composed of n-dimensional dynamical systems, each one $i = 1, \ldots, n$ characterized by $\dot{\boldsymbol{q}}_i = \boldsymbol{f}(\boldsymbol{q}_i)$. If we add diffusion along the chain, the behavior of cell i is characterized by:

$$\frac{d\boldsymbol{q}_i}{d\tau} = \boldsymbol{f}(\boldsymbol{q}_i) + \widetilde{D}(\boldsymbol{q}_{i+1} + \boldsymbol{q}_{i-1} - 2\boldsymbol{q}_i), \tag{1}$$

where $\widetilde{D}$ is the *diffusion tensor*. Now we consider a (stable) fixed point, $\boldsymbol{q}_0$, of the dynamical system, i.e. $\boldsymbol{f}(\boldsymbol{q}_0) = \boldsymbol{0}$. It also constitutes a fixed point for the entire chain since diffusion terms cancel ($\boldsymbol{q}_i = \boldsymbol{q}_j$ for all i and j). We want to analyze the effect of a small perturbation, $\boldsymbol{\Delta}$, around the steady state of the chain. Introducing the variables

$$\boldsymbol{q}_i = \boldsymbol{q}_0 + \boldsymbol{\Delta}_i, \tag{2}$$

into Equation 1, and expanding up to first order in $\boldsymbol{\Delta}$ one gets:

$$\frac{d\boldsymbol{\Delta}_i}{d\tau} = \nabla \boldsymbol{f}(\boldsymbol{q_0}) \cdot \boldsymbol{\Delta}_i + \widetilde{D}(\boldsymbol{\Delta}_{i+1} + \boldsymbol{\Delta}_{i-1} - 2\boldsymbol{\Delta}_i), \tag{3}$$

where $\nabla \boldsymbol{f}(\boldsymbol{q_0})$ is the Jacobian matrix of the field evaluated in the point $\boldsymbol{q}_0$. Furthermore, we can decompose the perturbation in terms of plane waves:

$$\begin{aligned} \boldsymbol{\Delta}_i(\tau) &= \sum_k \boldsymbol{\Delta}_{i,k}, \\ \boldsymbol{\Delta}_{i,k}(\tau) &= \boldsymbol{A} e^{\omega_k \tau} \cos(ki). \end{aligned} \tag{4}$$

The admissible values of the wavevector k depend on the length of the chain and on the boundary conditions. For instance, $k = n\pi/L$ with $n = 1, 2, 3, \ldots, L$ for Von Neumann (zero flux) boundary conditions. Introducing 4 into 3 we find:

$$\omega_k \boldsymbol{A} = \nabla \boldsymbol{f}(\boldsymbol{q_0})\boldsymbol{A} + 2\widetilde{D}\boldsymbol{A}(\cos k - 1), \tag{5}$$

which has nontrivial solutions if

$$\det(\nabla \boldsymbol{f}(\boldsymbol{q_0}) + 2\widetilde{D}(\cos k - 1) - \omega_k) = 0. \tag{6}$$

Therefore, the k mode is related to some possible frequencies ω_k. If those frequencies satisfy $\mathrm{Re}(\omega_k) < 0$, it is expected that the perturbation $\boldsymbol{\Delta}$ will be damped (negative exponential) and the

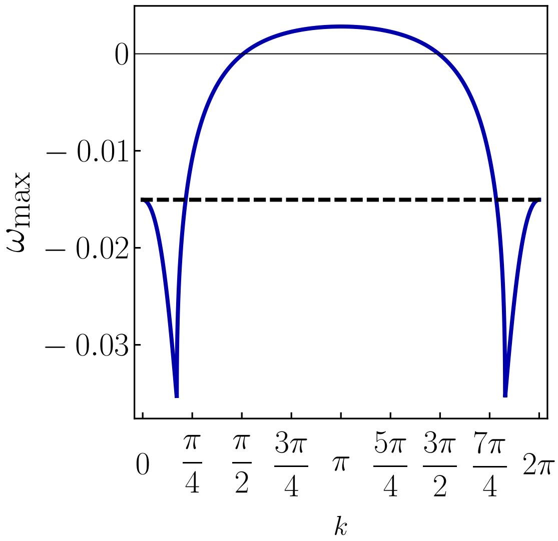

FIGURE 1: Results for $\omega_{\max}$ extracted from Equation 6 for the cyanobacterial system with $D_s = 0.1$ and $D_n = 0.2$ (Blue). The black dashed line indicates the value at equilibrium in the absence of diffusion

system will recover its initial equilibrium. Nevertheless, if $\mathrm{Re}(\omega_k) > 0$ perturbations will be amplified (positive exponential) and thus it is expected that structures of wavelength $2\pi/k$ will develop. The important value that determines if the system is stable to perturbations of some wavevector k is the largest real part of the ω_k from 6.

We can now turn to our cyanobacteria chain characterized by Eq. (16) of the main text. We analyze the effect of perturbations around the vegetative-like state for $D_s = 0.1$ and $D_n = 0.2$ (Figure 1). We find that the system is unstable against perturbations of intermediate wavevectors, with an upper bound (representing a minimum length at which patterns can be formed) and a lower bound (maximum length). The value of the minimum length (measured in cell number) $l_{\min} \approx 8/7 > 1$ so that a single cell is unable to differentiate.
