# Supplementary material for: Correction: An Integrative Approach for Modeling and Simulation of Heterocyst Pattern Formation in Cyanobacteria Filaments
Source: PLoS Comput Biol. 2015 Jul 8;11(7):e1004378. doi: 10.1371/journal.pcbi.1004378 (PMC4496046; doi:10.1371/journal.pcbi.1004378)
Supplement: S1 File — (PDF) [file pcbi.1004378.s001.pdf]

PLOS | COMPUTATIONAL BIOLOGY

RESEARCH ARTICLE

An Integrative Approach for Modeling and Simulation of Heterocyst Pattern Formation in Cyanobacteria Filaments

Alejandro Torres-Sánchez[1,2], **Jesús Gómez-Gardeñes**[1,3], **Fernando Falo**[1,3]*

1 Departamento de Física de la Materia Condensada, Universidad de Zaragoza, Zaragoza, Spain,
2 Laboratori de Càlcul Numèric, Universitat de Politècnica de Catalunya, Barcelona, Spain,
3 Instituto de Biocomputación y Física de Sistemas Complejos (BIFI), Universidad de Zaragoza, Zaragoza, Spain

* fff@unizar.es

CrossMark
click for updates

OPEN ACCESS

Citation: Torres-Sánchez A, Gómez-Gardeñes J, Falo F (2015) An Integrative Approach for Modeling and Simulation of Heterocyst Pattern Formation in Cyanobacteria Filaments. PLoS Comput Biol 11(3): e1004129. doi:10.1371/journal.pcbi.1004129

Received: July 21, 2014

Accepted: January 14, 2015

Published: March 27, 2015

Copyright: © 2015 Torres-Sánchez et al. This is an open access article distributed under the terms of the Creative Commons Attribution License, which permits unrestricted use, distribution, and reproduction in any medium, provided the original author and source are credited.

Data Availability Statement: All relevant data are within the paper and its Supporting Information files.

Funding: We acknowledge financial support from the Spanish MINECO under projects FIS2011-25167 and FIS2012-38266-C02-01. JGG is supported by the Spanish MINECO through the Ramon y Cajal program. The funders had no role in study design, data collection and analysis, decision to publish, or preparation of the manuscript.

Competing Interests: The authors have declared that no competing interests exist.

Abstract

Heterocyst differentiation in cyanobacteria filaments is one of the simplest examples of cellular differentiation and pattern formation in multicellular organisms. Despite of the many experimental studies addressing the evolution and sustainment of heterocyst patterns and the knowledge of the genetic circuit underlying the behavior of single cyanobacterium under nitrogen deprivation, there is still a theoretical gap connecting these two macroscopic and microscopic processes. As an attempt to shed light on this issue, here we explore heterocyst differentiation under the paradigm of systems biology. This framework allows us to formulate the essential dynamical ingredients of the genetic circuit of a single cyanobacterium into a set of differential equations describing the time evolution of the concentrations of the relevant molecular products. As a result, we are able to study the behavior of a single cyanobacterium under different external conditions, emulating nitrogen deprivation, and simulate the dynamics of cyanobacteria filaments by coupling their respective genetic circuits via molecular diffusion. These two ingredients allow us to understand the principles by which heterocyst patterns can be generated and sustained. In particular, our results point out that, by including both diffusion and noisy external conditions in the computational model, it is possible to reproduce the main features of the formation and sustainment of heterocyst patterns in cyanobacteria filaments as observed experimentally. Finally, we discuss the validity and possible improvements of the model.

Author Summary

Cyanobacteria filaments are paradigmatic examples of prokaryotic cellular differentiation and cooperative pattern formation. When a cyanobacteria filament is deprived of combined nitrogen, some vegetative cells differentiate into heterocysts, which are terminally differentiated nitrogen-fixing cells. Interestingly, most cells do not differentiate, but remain in their initial vegetative state. The coexistence of heterocysts and vegetative cells is

essential for the survival of the filament since (*i*) heterocysts lose their photosynthetic capacity so they need vegetative cells around to be provided with a source of fixed carbon and (*ii*) cell division, i.e. reproduction, is only accomplished by vegetative cells. From such a paradigmatic example it is clear that differentiation processes are the result of the interplay of complex regulatory networks acting inside the cell and external stimuli, coming from both the adjacent cells and the environment. In this article we present an integrative approach that combines the study of internal regulatory processes, diffusion, and noisy environments in order to capture the key mechanisms leading to the differentiation of vegetative cyanobacteria into heterocysts and the subsequent pattern formation.

Introduction

The formation of multicellular organisms from the assembly of single-celled ones constitutes one of the most striking and complex problems tackled by biology. The most salient feature that characterizes multicellular organisms is the presence of different cell types, in such a way that the organism associates a different function to each cell type. In each of these cellular types, only a subset of the genes that constitute the genome of the organism (genotype) are expressed, which identify the function and morphology of the cell (phenotype). The development of specialized cells involves differentiation processes, which lead to alterations in gene expression producing different phenotypes from a given genotype. These processes are highly dynamical, directed by complex regulatory networks involving cell-to-cell interactions, and often triggered by external stimuli. As a result of the differentiation processes a rich cooperative pattern involving different cell types is established, increasing the complexity and adaptability of the organism. Due to the large number of scales involved, ranging from protein binding to diffusion of specific elements throughout the organism, a correct mathematical modeling of differentiation processes and their associated pattern formation demands an integrative approach combining tools from statistical mechanics and the theory of dynamical systems (see [1, 2] for instance).

A landmark process of (prokaryotic) cellular differentiation and cooperative pattern formation is the heterocyst differentiation in cyanobacteria filaments [3, 4]. Cyanobacteria are one of the first organisms that developed multicellularity some (2–3) billion years ago [5]. These bacteria perform oxygenic photosynthesis releasing oxygen to the environment. However, nitrogenase, the enzyme that performs nitrogen fixation, is deactivated by oxygen so that nitrogen fixation cannot occur in its presence [6]. Cyanobacteria solve the incompatibility of incorporating both oxygenic photosynthesis and nitrogen fixation by separating these processes (*i*) temporally, such as in the unicellular *Cyanothece sp.* strain ATCC 51142, which presents photosynthetic activity during the day and fixes nitrogen during the night [7], or (*ii*) spatially, by the generation of non-photosynthetic nitrogen-fixing cells distributed along the filament and acting as nitrogen suppliers.

In the presence of combined nitrogen (such as nitrate, nitrite, ammonium or urea), most cyanobacteria (*Anabaena* PCC strain 7120 being the most representative example) form long filaments of photosynthetic vegetative cells. However, in the absence of combined nitrogen (cN), a subset of the vegetative cells differentiate into heterocysts, which are terminally differentiated nitrogen-fixing cells. By differentiating, heterocysts lose their photosynthetic capacity, so they require an external source of fixed carbon [8, 9]. To this aim, each forming heterocyst sends a signal, by means of the production of some substance that diffuses along the filament, to prevent the differentiation of its neighboring cells. A cooperative pattern is thus established:

heterocysts provide cN to the filament while vegetative cells supply fixed carbon. As a result, heterocysts appear interspersed with around 10 vegetative cells, depending on the species, forming a semi-regular pattern that remains approximately constant regardless of cell division [10, 11]. The resulting pattern forms one of the simplest and most primitive examples of a multicellular organism as a product of the interdependence between heterocysts and vegetative cells. Interestingly, an isolated cyanobacterium does not differentiate but it first divides so that one of the descendants differentiates. This latter mechanism is crucial since (*i*) a sole heterocyst would lack a source of fixed carbon and (*ii*) it would not reproduce as it is a terminally differentiated cell [12].

Let us briefly review the previous studies on the mathematical modeling of heterocyst pattern formation. In references [13, 14] Rutenberg and coworkers analyzed a model to explain heterocyst patterns by means of the study of cN diffusion along a cyanobacterial filament. On the other hand, Gerdtzen *et al.* [15] modeled cyanobacterial filaments based on a time-discrete dynamical system incorporating the main interactions between the most important proteins that take part in heterocyst formation.

In this work, we develop a simple mathematical model by incorporating the recent experimental results on the genetic regulatory network of cyanobacteria into the theoretical machinery of system biology.

Our model connects the diffusion of combined nitrogen along the filament with the dynamical properties of the underlying genetic circuit of each single cyanobacterium, capturing both the development of heterocyst patterns and their maintenance. Furthermore, our model shows that noise plays an important role in the onset of differentiation by enabling the development of the characteristic heterocyst patterns for a wide range of model parameters. This reveals that cyanobacteria filaments have developed an efficient response to the noisy conditions that characterize the natural environment.

The work is structured as follows. First we present the main actors of the basic regulatory network and the different dynamical interactions that take place during the differentiation process. Then we develop a mathematical model for the unicellular reaction to nitrogen deprivation. Although a single cell model cannot provide a complete understanding of heterocyst formation, we analyze the main features that arise from the dynamical behavior of the system to gain insight about cell dynamics under different external conditions.

Finally, we round off the paper by introducing the spatial model consisting of a filament of cyanobacteria, each one characterized by the dynamical circuit developed previously, that interact by means of protein diffusion.

Results

Description of the main genes and their basic genetic circuit

Heterocyst development begins with sensing combined-nitrogen (cN) limitation and ends with nitrogen fixation in mature heterocysts. This process is usually completed after 20 hours at 30°C [9]. In Fig. 1 we show a basic scheme of the genetic circuit including the most relevant elements and their respective interactions. Here we explain the main features of this genetic circuit.

The process is initiated with the accumulation of 2-oxoglutarate (2-OG) as a consequence of cN deprivation [9, 16]. 2-OG interacts with ammonium through the GS/GOGAT cycle [17–19] (see Fig. 2). Under cN starvation, the GS/GOGAT cycle breaks down, leading to the accumulation of 2-OG inside the cell [9]. In its turn, 2-OG stimulates the DNA-binding activity of NtcA, an important transcription factor for heterocyst development [18, 20, 21]. Furthermore, the transcription of the genes targeted by NtcA does not start in the absence of 2-OG [22, 23].

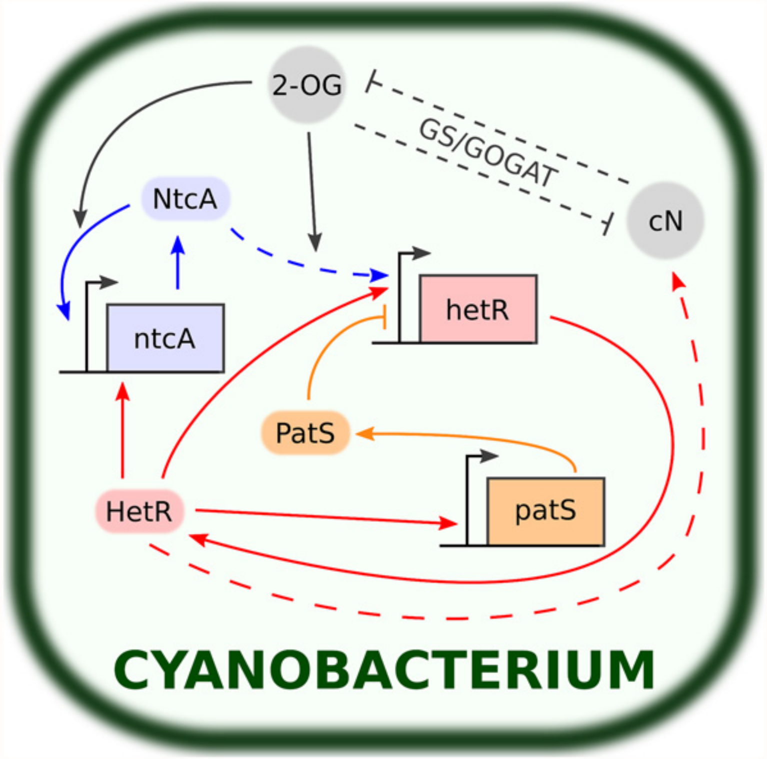

Fig 1. Main components and interactions involved in the reaction to combined nitrogen deprivation in cyanobacteria. Rectangular boxes represent genes (*ntcA, hetR* and *patS*) while rounded boxes and circles represent transcription factors (NtcA, HetR and PatS) and smaller molecules (2-OG and cN) respectively. Normal-tipped and flat-tipped arrows stand for up-regulating and down-regulating processes respectively. Dashed lines stand for indirect or imperfectly understood interactions. The accumulation of 2-OG enhances the DNA-binding activity of NtcA, which in turn up-regulates the transcription of *ntcA* and *hetR*. HetR activates *ntcA* and *hetR* (composing the central NtcA-HetR autoregulatory loop), the inhibitor *patS* and other genes that lead to nitrogen fixation and the morphological changes involved in heterocyst differentiation. 2-OG and cN levels are linked through the GS/GOGAT cycle (see Fig. 2).

doi:10.1371/journal.pcbi.1004129.g001

NtcA presents autoregulation [22, 24, 25] and indirectly activates the key gene that controls cell differentiation and pattern formation: *hetR* [26–28]. To bind DNA, NtcA needs to homodimerize [29, 30]. In conclusion, the accumulation of 2-OG is the factor that triggers differentiation. In agreement with this idea, artificial increased levels of 2-OG result in heterocyst development even in the presence of ammonium [16, 18, 31].

The next step in heterocyst development is the activation of *hetR*. Remarkably, null mutants of *hetR* do not produce heterocysts whereas an overexpression of *hetR* leads to an increased heterocyst frequency [27, 32, 33]. The transcription of *hetR* is induced by NtcA through the action of an intermediate, *nrrA* [28]. The DNA-binding activity of HetR requires its homodimerization [34, 35]. Multiple transcription factors related to heterocyst formation are up-regulated by HetR, including *hetR* itself [35], *ntcA* [36] and *patS* [35].

The up-regulatory loop of NtcA and HetR is essential for heterocyst differentiation [36–39]. However, the action of NtcA and HetR alone cannot explain pattern formation. Another transcription factor, PatS, inhibits the DNA-binding activity of HetR [8, 35, 40, 41]. This inhibitory behavior is essential for the communication with adjacent cells and thus to achieve the observed patterns of vegetative cells and heterocysts in cyanobacteria filaments (see Fig. 3). Furthermore, *patS* is strongly expressed in differentiating cells and mature heterocysts due to its

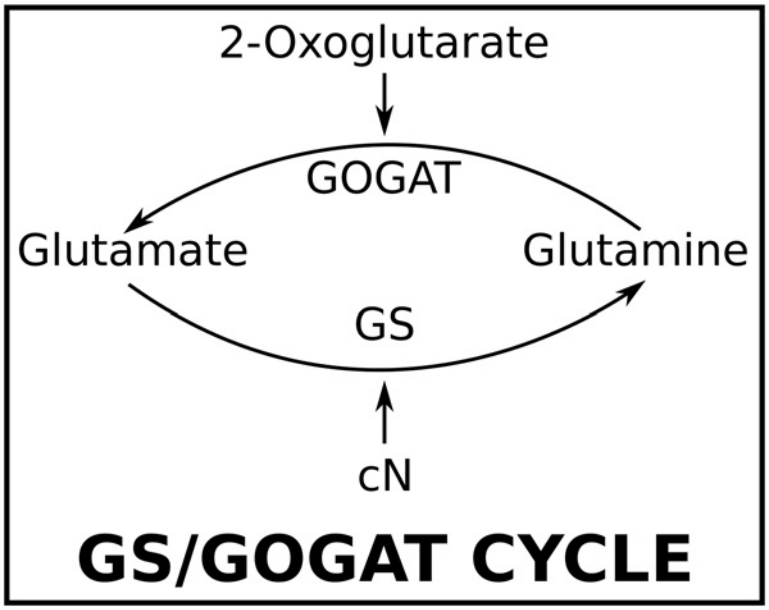

Fig 2. GS/GOGAT cycle. 2-OG and cN indirectly interact through the GS/GOGAT cycle. Glutamine is transformed into glutamate by means of 2-OG through the 2-OG amidotransferase (GOGAT) while cN converts glutamate into glutamine through the glutamine synthetase (GS). The importance of the cycle in heterocyst differentiation is twofold. From one side, it constitutes the early one-cell sensor to nitrogen starvation: the absence of cN breaks the cycle down and 2-OG starts to accumulate, whose action leads to the cascade of processes that provoke the differentiation (see Fig. 1). Additionally, later during the differentiation, it processes the cN created by the heterocysts decreasing the levels of 2-OG. The latter is crucial for the formation of the heterocyst pattern (see Fig. 3).

doi:10.1371/journal.pcbi.1004129.g002

upregulation by HetR [8]. A filament without *patS* develops multiple contiguous heterocysts (about a 30% of all cells as compared to the usual 10% in the wild-type filament). On the other hand, an over-expression of *patS* suppresses heterocyst differentiation [9]. Moreover, the addition to the growth medium of a synthetic peptide composed of the last five residues (RGSGR) of PatS (PatS5) inhibits heterocyst development, suggesting that PatS5 may be a diffusive mature form of PatS that stops the differentiation of the rest of vegetative cells of the filament

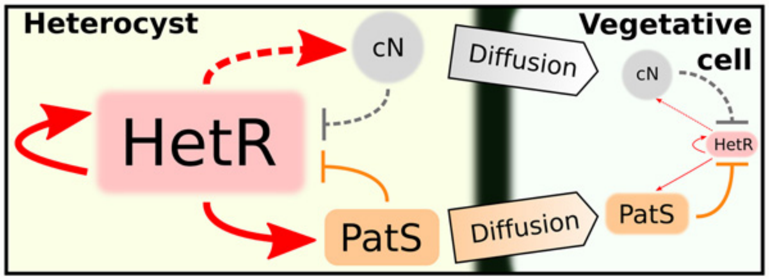

Fig 3. Diffusion scheme. Schematic representation of the diffusion processes that sustain the heterocyst pattern. Heterocysts produce cN and PatS. cN diffuses along the filament where, due to the action of the GS/GOGAT cycle (see Fig. 2), decreases the levels of 2-OG breaking the autoregulatory core NtcA-HetR. Early during the differentiation, PatS (or other derivative of it, see the text) diffuses along the filament inhibiting HetR. Both processes combined prevent the differentiation of the rest of vegetative cells and explain the formation of the pattern.

doi:10.1371/journal.pcbi.1004129.g003

[40]. A similar protein carrying the RGSGR pentapeptide, with a similar effect as that of PatS, is HetN [42]. A chain lacking both PatS and HetN leads to a lethal phenotype in which all cells differentiate [43].

The last stages of heterocyst development cause the physiological changes of the cell aimed at creating an anaerobic environment that sustains nitrogen fixation. To this end, two new membrane layers are biosynthesized to decrease the entry of oxygen into the cell [44]. The morphogenesis of these two layers is controlled by two family of genes, *hep* and *hgl*, that are indirectly up-regulated by HetR [35]. After these morphological changes the genes in charge of nitrogen fixation, *nif* genes, are expressed. These genes encode, among others, the enzyme nitrogenase, which ultimately performs nitrogen fixation.

The fixed nitrogen of the new heterocysts acts as an inhibitor of the differentiation together with PatS and HetN [45]. Thus, the diffusion of these inhibitors from heterocysts along the filament plays a key role in pattern maintenance (see Fig. 3). As a result of the differentiation heterocysts produce fixed nitrogen from N_2 of the atmosphere and they interchange this nitrogen with the oxygen derivatives produced by the vegetative cells, in an illustration of the cooperative behavior between cell types in a multicellular organism.

Regulatory equations

In this section we translate the genetic circuit previously described into a set of differential equations, for which we follow the derivation in [46–48]. Details are left to supplementary information (S1 text). To simplify notation, constants related to NtcA, HetR, PatS and cN are denoted with the letters *a, r, s*, and *n* respectively.

We start by looking at the transcription of *ntcA*, which is regulated by HetR and NtcA. We assume that the probability that NtcA binds the promoter in the absence of 2-OG can be neglected. Taking into account that both HetR and NtcA dimerize to bind DNA we find:

$$v_a = L_a + \frac{v_a^a \kappa_a^a [\text{2-OG}][\text{NtcA}]^2 + v_a^r \kappa_a^r [\text{HetR}]^2 + v_a^{ar} \kappa_a^a \kappa_a^r [\text{2-OG}][\text{NtcA}]^2 [\text{HetR}]^2}{(1 + \kappa_a^a [\text{2-OG}][\text{NtcA}]^2)(1 + \kappa_a^r [\text{HetR}]^2)}, \quad (1)$$

where v_a measures the production rate of NtcA in units of concentration per time, v_a^a, v_r^a and v_{ar}^a are the rates when only NtcA, only HetR or both are bound to DNA respectively, and κ_*^a are the inverse of the effective dissociation constants of the compounds that bind DNA. L_a, the so-called leak term, measures the basal production of *ntcA* in the absence of regulation. Subscripts and superscripts identify the binding site and the transcription factor for which the constants are given respectively.

Similarly we can obtain the transcription velocity for HetR. We assume that *hetR* is regulated by NtcA by means of a usual Hill function, yet the real process presents an intermediate, *nrrA*. To do so, we take into account that *nrrA* concentration relaxes rapidly to a limiting value. Furthermore, PatS affects the auto-regulatory loop of HetR. It has been suggested that PatS binds the binding site of HetR in the promoter of *hetR* preventing HetR binding [35]. These facts, along with the influence of 2-OG levels, provide with an expression for the transcription velocity:

$$v_r = L_r + \frac{v_r^a \kappa_r^a [\text{2-OG}][\text{NtcA}]^2 (1 + \kappa_r^s [\text{PatS}]) + v_r^r \kappa_r^r [\text{HetR}]^2 + v_r^{ar} \kappa_r^a \kappa_r^r [\text{2-OG}][\text{NtcA}]^2 [\text{HetR}]^2}{\left(1 + \kappa_r^a [\text{2-OG}][\text{NtcA}]^2\right)\left(1 + \kappa_r^r [\text{HetR}]^2 + \kappa_r^s [\text{PatS}]\right)} \quad (2)$$

HetR regulates most processes of the genetic circuit. It governs, among others, the transcription of *ntcA, patS, hep, hgl* and *nif* genes that lead to most of structural changes of the cell and to nitrogen fixation.

The inhibitor PatS is regulated by HetR, and we assume no other influence. For simplicity, we implicitly include the effect of HetN in PatS, as their action is expected to be equivalent (see the previous section). This gives the simple transcription velocity:

$$v_s = L_s + \frac{v_s^r \kappa_s^r [\mathrm{HetR}]^2}{1 + \kappa_s^r [\mathrm{HetR}]^2} \tag{3}$$

Finally, we have to relate nitrogenase concentration [Ni] to that of combined Nitrogen [cN], both regulated by HetR and the levels of 2-OG [2-OG]. Let us begin by examining nitrogenase concentration, which is directly controlled by *nif* genes. Although this is not a direct process, we can assume, as we did for the NtcA-regulation of *hetR*, that *nif* genes are functionally governed by [HetR] following a typical Hill function. The nitrogenase production rate is given by:

$$\frac{d[\mathrm{Ni}]}{dt} = L_{Ni} + \frac{v_{Ni}^r \kappa_{Ni}^r [\mathrm{HetR}]^2}{1 + \kappa_{Ni}^r [\mathrm{HetR}]^2} - \delta_{Ni}[\mathrm{Ni}]. \tag{4}$$

where δ_{Ni} represents the degradation rate of nitrogenase. We can effectively account for the lag introduced by intermediate processes not taken into account explicitly in the model by increasing the value of δ_{Ni} so that [Ni] relaxes more slowly. Assuming that nitrogenase produces fixed nitrogen at a constant rate, we arrive at the equation that governs cN levels in cyanobacteria:

$$\frac{d[\mathrm{cN}]}{dt} = L_n' + v_n'[\mathrm{Ni}] - \delta_n'[\mathrm{cN}], \tag{5}$$

where L_n' represents the flux of cN from the exterior of the cell. Assuming that the levels of cN relax rapidly we solve Eq. (5) for the steady state. Substituting in Eq. (4) we find:

$$\frac{d[\mathrm{cN}]}{dt} = L_n + \frac{v_n^r \kappa_n^r [\mathrm{HetR}]^2}{1 + \kappa_n^r [\mathrm{HetR}]^2} - \delta_n[\mathrm{cN}]\ , \tag{6}$$

where

$$L_n = \frac{1}{\delta_n'}\left(v_n' L_{\mathrm{Ni}} + \delta_{\mathrm{Ni}} L_n'\right), \qquad v_n^r = \frac{v_n'}{\delta_n'} v_{\mathrm{Ni}}^r, \qquad \delta_n = \delta_{\mathrm{Ni}}, \qquad \kappa_n^r = \kappa_{Ni}^r. \tag{7}$$

To get a closed system of equations, we shall investigate the relation between cN and 2-OG. Both are related by means of the GS/GOGAT cycle (Fig. 2). Assuming the cycle is in equilibrium and reactions are grounded on the law of mass action, the following two conditions must be satisfied:

$$[\mathrm{glutamate}] = \kappa_{\leftarrow}[\mathrm{glutamine}][\,2\text{-OG}\,], \qquad [\mathrm{glutamine}] = \kappa_{\rightarrow}[\mathrm{glutamate}][\mathrm{cN}], \tag{8}$$

which lead to the relation:

$$[\,2\text{-OG}\,] = \frac{1}{\kappa_{\leftarrow}\kappa_{\rightarrow}[\mathrm{cN}]}. \tag{9}$$

However, this expression does not behave properly for small concentrations of cN, which are expected under cN deprivation: 2-OG levels would increase without limit. In fact, 2-OG production is controlled by some processes that are not considered in this work and so its value must be limited. We can effectively include such a limiting value by means of a translation on

[cN] in Eq. (9)

$$[\text{2-OG}] = \frac{1}{\kappa_{\text{2-OG}} + \kappa_{\leftarrow}\kappa_{\rightarrow}[\text{cN}]}, \tag{10}$$

which reaches the maximum value $[\text{2-OG}]_{\max} = 1/\kappa_{\text{2-OG}}$ at $[\text{cN}] = 0$.

Finally we introduce the differential equations governing cyanobacterial reaction to nitrogen deprivation. They represent the temporal variation of the most important factors of the genetic circuit, namely NtcA, HetR, PatS and cN. Using the production rates (1), (2), (3), (6) and introducing degradation rates constants, δ_*, we find:

$$\begin{aligned}
\frac{dq_a}{d\tau} &= l_a + \frac{\beta_a^a\gamma_a^a q_a^2 + \beta_a^r\gamma_a^r q_r^2(1+q_n) + \beta_a^{ar}\gamma_a^a q_a^2\gamma_a^r q_r^2}{(1+q_n+\gamma_a^a q_a^2)(1+\gamma_a^r q_r^2)} - d_a q_a,\\
\frac{dq_r}{d\tau} &= l_r + \frac{\beta_r^a q_a^2(1+q_s) + \beta_r^r q_r^2(1+q_n) + \beta_r^{ar} q_a^2 q_r^2}{(1+q_n+q_a^2)(1+q_s+q_r^2)} - q_r,\\
\frac{dq_s}{d\tau} &= l_s + \frac{\beta_s^r\gamma_s^r q_r^2}{1+\gamma_s^r q_r^2} - d_s q_s,\\
\frac{dq_n}{d\tau} &= l_n + \frac{\beta_n^r\gamma_n^r q_r^2}{1+\gamma_n^r q_r^2} - d_n q_n,
\end{aligned} \tag{11}$$

where we have introduced the dimensionless variables:

$$q_a = \underbrace{\sqrt{\frac{\kappa_r^a}{\kappa_{\text{2-OG}}}}}_{\phi_a}[\text{NtcA}], \quad q_r = \underbrace{\sqrt{\kappa_r^r}}_{\phi_r}[\text{HetR}], \quad q_s = \underbrace{\kappa_r^s}_{\phi_s}[\text{PatS}],$$
$$q_n = \underbrace{\frac{\kappa_{\leftarrow}\kappa_{\rightarrow}}{\kappa_{\text{2-OG}}}}_{\phi_n}[\text{cN}], \qquad \tau = \delta_r t, \tag{12}$$

and the constants

$$l_* = \frac{L_*\phi_*}{\delta_r}, \qquad \beta_*^\bullet = \frac{\nu_*^\bullet\phi_*}{\delta_r}, \qquad \gamma_*^\bullet = \frac{\kappa_*^\bullet}{\kappa_r^\bullet}, \qquad d_* = \frac{\delta_*}{\delta_r}. \tag{13}$$

Let us finally stress that this is a *deterministic* model for a single cyanobacterium. The study of the cyanobacterial filament is left to the final section. We show that the main modification will be adding diffusion processes for the inhibitors PatS and cN through the chain. An important ingredient in pattern formation, noise, will be also added to the equations.

Unicellular dynamics

In this section we analyze the dynamical system (11) for a set of constants (Table 1) that exhibit both the dynamical and the structural properties of heterocyst differentiation. Following the usual practice in the analysis of dynamical systems, we study the basic properties of equations (11), such as fixed points and linear stability analysis, to analyze the key features leading to heterocyst differentiation.

Taking into account the difference between the relaxation times of the constituents of the model, given by the inverses of d_* (see Table 1), we can interpret it as composed of two temporally separated systems: a rapid one, formed by HetR and NtcA, showing fast dynamics that relaxes to its steady state almost instantaneously and a slow one, composed of PatS and cN, whose evolution is dictated by the values of HetR and NctA in their instantaneous equilibrium.

Table 1. Parameters for Eq. (11) that reproduce heterocyst formation under noisy conditions and pattern formation when PatS and cN diffuse along a filament of cyanobacteria.

Constants			
$l_a = 0.2$	$l_r = 0.01$	$l_s = 0.0001$	$l_n = 0$
$d_a = 0.7$	$d_s = 0.05$	$d_n = 0.01$	$\beta_a^a = 4$
$\beta_a^r = 4$	$\beta_a^{ar} = 8$	$\beta_r^a = 1$	$\beta_r^r = 1$
$\beta_a^{ar} = 3$	$\beta_s^r = 0.385$	$\beta_n^r = 0.06$	$\gamma_a^a = 3$
$\gamma_a^r = 2.4$	$\gamma_s^r = 1.2$	$\gamma_n^r = 2.75$	

doi:10.1371/journal.pcbi.1004129.t001

This corresponds to an adiabatic elimination technique [49] that helps in reducing the complexity of the dynamical system by splitting it into two simpler interdependent subsystems.

First, we look at the fixed points of q_a and q_r for each pair of values of q_s and q_n

$$f_a(q_s, q_n) = \frac{dq_a}{d\tau} = 0, \qquad f_r(q_s, q_n) = \frac{dq_r}{d\tau} = 0. \tag{14}$$

The numerical solution to this problem is sketched in Fig. 4. We find three different branches of solutions that coexist in some regions. The fixed points on the lower and upper branches are always stable (blue region in Fig. 4I) and those lying on the middle branch (red

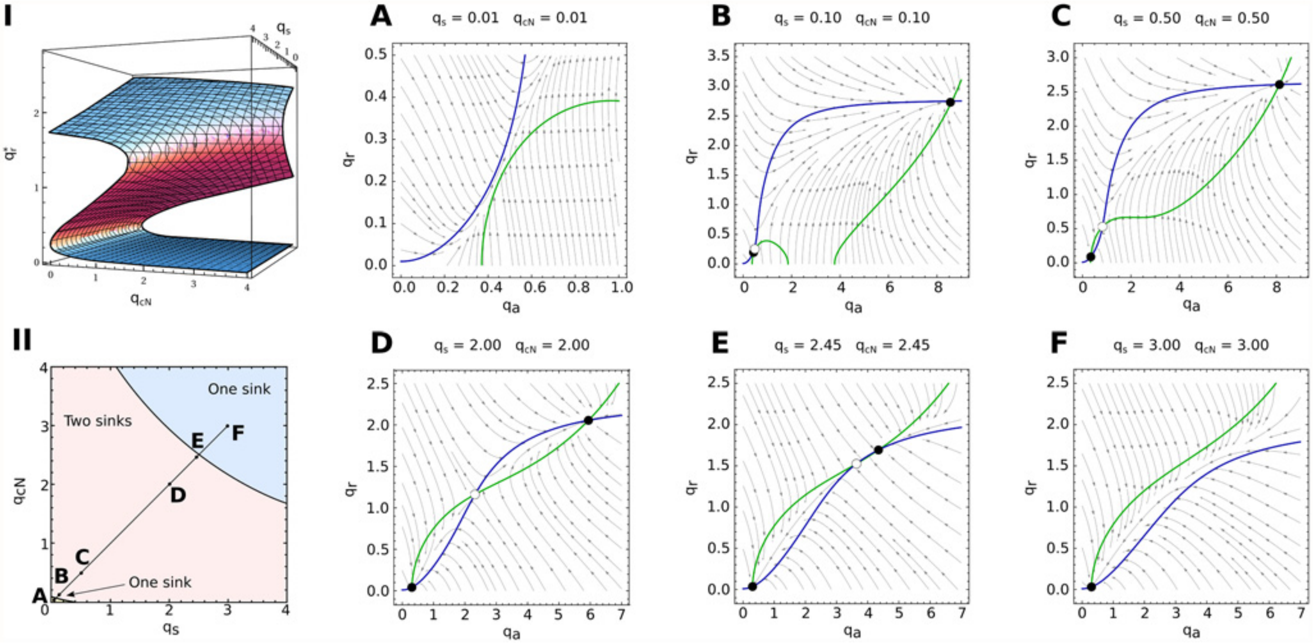

Fig 4. Adiabatic elimination of the fast variables q_r and q_a. Due to the fast dynamics that HetR and NtcA exhibit, we can approach the treatment of the system by adopting a point of view that follows the slower variables q_s and q_n. From this viewpoint, the time-evolution of the pair ($q_s(t)$, $q_n(t)$) is considered by assuming that q_r and q_a instantaneously relax to an equilibrium, which corresponds to a sink (q_r^*, q_a^*) for the fixed pair ($q_s(t)$, $q_n(t)$). Depending on the region of the (q_s, q_n)-plane, there are three fixed points (two sinks corresponding to the highest and the lowest concentrations respectively and a saddle in the middle) or one (a sink) for q_r and q_a (I and II). There are two one-sink regions that are separated from the two-sink region by saddle-node bifurcations (A-F). Sinks and saddles are represented by filled and unfilled circles respectively and arrows indicate the flow of the dynamics. We can then imagine the dynamics of q_s and q_n as evolving either in the bottom or in the top branch of I. In the two-sink region, both branches are plausible and the history of the dynamics determine the solution (hysteresis effect): a dynamics in a branch will continue in it until experiencing a bifurcation in the (q_r, q_a) plane (see Fig. 5 for examples).

doi:10.1371/journal.pcbi.1004129.g004

region) are saddles. Transitions between the regions with one and three fixed points correspond to saddle-node bifurcations in which the middle branch of solutions coalesce with the lower and the upper one respectively. The basins of attraction of both stable fixed points are separated by the stable manifold of the saddle point (Fig. 4II).

In the bistable region the system behaves as a switch that can be either OFF in a vegetative state (lower branch, with a small production of HetR and NtcA) or ON in a heterocyst state (upper branch, with a high production of HetR and NtcA). A sufficient large perturbation may result in the system crossing the manifold of the saddle and falling into the other stable branch of solutions. The distance between the saddle and the nodes determines the size of the perturbation needed to activate or inactivate the system.

With (14) solved, we can apply the solution to calculate the effective field sensed by the (q_s, q_n) pair. In the regions showing bistability the field takes two very different forms, one corresponding to the values of the lower branch and another corresponding to those of the upper one (Fig. 5). We expect a *hysteresis effect*: if initially the dynamics lies on a particular branch it will remain on it unless a fluctuation or a bifurcation makes the system jump to the other branch.

In the presence of cN (Fig. 5A) we find only one stable fixed point that corresponds to a vegetative state (lower branch). The upper branch is completely unstable: any dynamics lying on it will fall down to the lower branch and eventually be attracted to the vegetative sink. The steady state is very robust against perturbations since it is far from the bifurcation region and there is a significant distance to the saddle in the $q_r - q_a$ plane.

By reducing the flow of cN from the exterior of the cell ($l_n = 0$) we find that a stable fixed point appears in the upper branch, a heterocyst state, while the vegetative state gets closer to

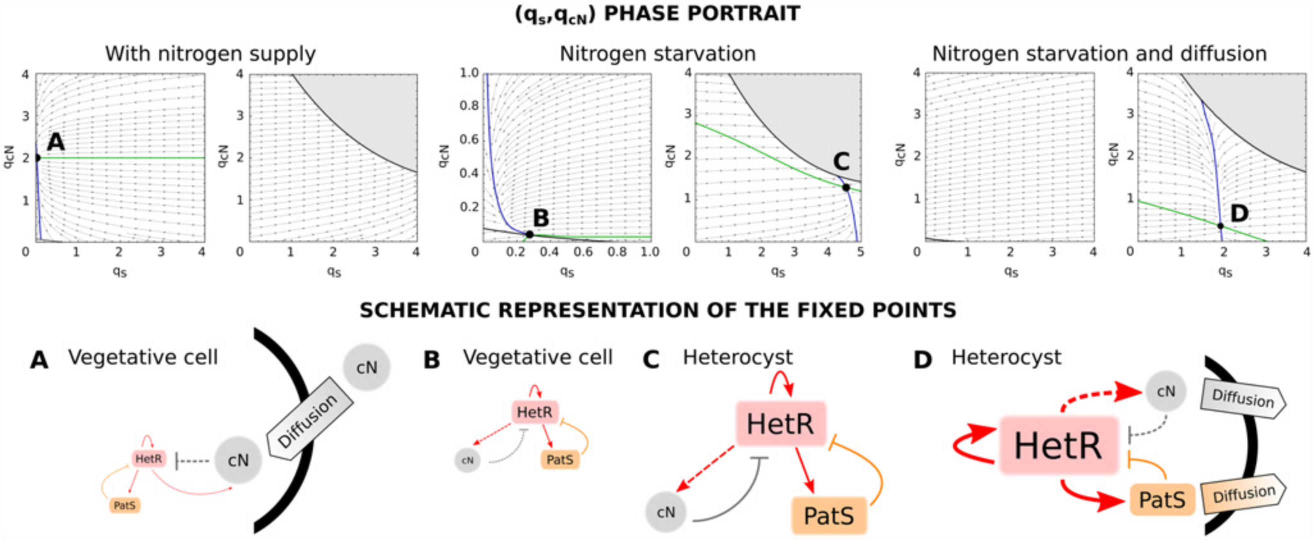

Fig 5. States of a cyanobacterium when subjected to different conditions of nitrogen and diffusion. When the cell is provided of cN ($l_n = 0.03$), there is only one stable fixed point (A) in the bottom branch, which corresponds to a state in which the production of both HetR and PatS is minimum (vegetative state). When subjected to nitrogen deprivation ($l_n = 0$), there are two stable fixed points (B and C) each one in a different branch. The first point (B) is a vegetative state in which there exists an equilibrium between a small production of HetR, PatS and cN. The same kind of equilibrium is present in the second fixed point (C) but in this case the production of all TFs and cN is high (heterocyst steady state). When the cell is exposed to nitrogen stress its trajectory evolves from A to the steady state B and thus it remains vegetative. Assuming some diffusion of cN and PatS from the cell ($l_s = -0.2$ and $l_n = -0.002$), the only stable state (D) corresponds to a heterocyst state with high levels of production of HetR, cN and PatS, being the latter transported to the surroundings of the cell.

doi:10.1371/journal.pcbi.1004129.g005

the bifurcation region, thus becoming more susceptible to perturbations that can make the system reach the upper branch. In the absence of cN, the cyanobacterium would evolve from state A to state B in the lower branch until a perturbation pushes it to the upper branch, eventually becoming an heterocyst due to the field acting on that branch (Fig. 5B and C).

Diffusion protects cells in the neighborhood of the newly formed heterocyst to initiate the differentiation: as heterocysts are producers of cN and PatS, the vegetative fixed point of the cells in its neighborhood will move towards an A-like state, thus becoming more stable to perturbations. The heterocyst fixed point also becomes more stable due to diffusion, since its production of inhibitors is distributed among other cells (see Fig. 5D).

Strains of cyanobacteria. Heterocyst patterns

In the previous section, we introduced a single cell model for the cyanobacteria reaction to nitrogen-limiting conditions. There we have shown that, for a specific range of parameters, the model exhibits features that would lead to heterocyst development under noisy conditions. Nevertheless, the model should be extended to cyanobacteria chains to account for heterocyst development since, as previously noted, isolated cyanobacteria do not become heterocysts by themselves; the action of the chain is needed to generate heterocysts.

In this section, we extend the previous results and consider a chain of vegetative cells facing nitrogen deprivation. The main modification is the introduction of diffusion of PatS and cN along the cyanobacteria chain. For this purpose we add to Eq. (12) the discrete version of the diffusion equation:

$$\frac{dC_i}{dt} = D_C\left(C_{i+1} + C_{i-1} - 2C_i\right). \tag{15}$$

where D_C is called the *diffusion constant* of the element C. Now, it is straightforward to introduce PatS and cN diffusion into the equations. The dynamics of cell i is characterized by the following set of equations:

$$\begin{aligned}
\frac{dq_{i,a}}{d\tau} &= l_a + \frac{\beta^a_a\gamma^a_a q_a^2 + \beta^r_a\gamma^r_a q_r^2(1+q_n) + \beta^{ar}_a\gamma^a_a q_a^2\gamma^r_a q_r^2}{(1+q_n+\gamma^a_a q_a^2)(1+\gamma^r_a q_r^2)} - d_a q_{i,a} + G_{i,a}(t),\\
\frac{dq_{i,r}}{d\tau} &= l_r + \frac{\beta^a_r q_a^2(1+q_s) + \beta^r_r q_r^2(1+q_n) + \beta^{ar}_r q_a^2 q_r^2}{(1+q_n+q_a^2)(1+q_s+q_r^2)} - q_{i,r} + G_{i,r}(t),\\
\frac{dq_{i,s}}{d\tau} &= l_s + \frac{\beta^r_s\gamma^r_s q_r^2}{1+K^r_s q_r^2} - d_s q_{i,s} + D_s(q_{i+1,s} + q_{i-1,s} - 2q_{i,s}) + G_{i,s}(t),\\
\frac{dq_{i,n}}{d\tau} &= l_n + \frac{\beta^r_n\gamma^r_n q_r^2}{1+\gamma^r_n q_r^2} - d_n q_{i,n} + D_n(q_{i+1,n} + q_{i-1,n} - 2q_{i,n}) + G_{i,n}(t),
\end{aligned} \tag{16}$$

which constitutes the model for a cyanobacteria filament. To account for environment variability we add white noise, $G_{i,*}(t)$, of the same amplitude, $\langle G_{i,*}(t)G_{i,*}(t')\rangle = \xi\delta(t-t')$, for all the components of the system. Based on these equations, we investigate the conditions that lead to a heterocyst pattern. It is easy to notice that they correspond to an activator-inhibitor system of cells coupled in a reaction-diffusion scheme [50]. This kind of system produces regular pattern formation [51–53]. Turing (linear stability) analysis of equations (16) (see S2 text) provides insight on the periodicity of patterns. It is interesting to show that the minimum periodicity observed in such analysis is larger than 1, which means that a single bacteria is unable to differentiate.

We performed the direct integration of equations (16) for chains of 200 cyanobacteria. We used a Runge-Kutta method for the numerical integration of stochastic differential equations

(see Methods) [54]. All simulations were performed with periodic boundary conditions, i.e. emulating a circular filament, for simplicity. We have also tested the more realistic no flux boundary conditions and find no change in gene dynamics and heterocyst patterns in the interior of the filament. This shows that the effect of boundary conditions is highly localized around the borders. In simulations with no flux boundary conditions no heterocysts were found in the border, which is in good agreement with experimental observations [55]. The level of noise that best reproduces heterocyst pattern is $\xi = 0.001$ for the set of parameters of Table 1. Importantly, isolated cells do not initiate differentiation with this level of noise, in agreement with the results from the linear stability analysis. Diffusion constants have been set to $D_s = 0.1$ and $D_n = 0.2$. Heterocysts patterns develop for different levels of noise and diffusion constants, but the model parameters, which characterize cell response to nitrogen deprivation, should change accordingly. This correlation between noise, diffusion and model parameters supports the idea that cyanobacteria have evolved towards a better response to the normal levels of noise in their environment.

In Fig. 6 we show the dynamics that the 4 variables exhibit when the filament is deprived of cN. We observe that the filament concentrations relax to the constant protein levels of the vegetative state we showed in the previous chapter. Then, due to the coupled action of noise and diffusion, some cells start to differentiate. As new forming heterocysts appear, their production and exportation of inhibitors to the surrounding cells make the latter more stable to perturbations stopping their differentiation. The model reproduces very well the initial peak that both NtcA and HetR present experimentally [35, 56]. PatS increases more slowly to its steady value reducing the levels of NtcA and HetR and, finally, cN is generated by heterocysts stabilizing the pattern.

Fig. 7 shows the evolution of the profile for a 200 cells chain of cyanobacteria. We observe that heterocysts progressively appear in those regions in which other heterocysts do not have effect (*i.e.* those vegetative cells that are not supplied of sufficient cN and PatS). Finally, a semi-regular pattern is generated. PatS and cN diffuse along the filament exhibiting smooth variations between vegetative cells and heterocysts, while HetR and NtcA present very abrupt variations between cell types.

Finally, in Fig. 7B we show the time-evolution of the histogram for the distance between two consecutive heterocysts. It should be stressed that although initially some close heterocysts appear, they are eliminated by the non-linear action of the system during the differentiation process. Close heterocysts compete for the same region of action (the same vegetative cells that consume their PatS and cN) and then they cannot reach the optimal heterocyst state, which is stable to slight perturbations. Finally, one of them falls down from the upper branch becoming a vegetative cell. This behavior is typically observed experimentally [4, 9]. The final histogram can be nicely fitted by a Γ-distribution, implying Poisson-distributed wait times for the underlying noise driven process.

Discussion

The study of cell differentiation and its underlying mechanisms constitutes one of most intriguing problems in biology. This phenomenon is the basis for multicellular organism and pattern formation. The approach presented here deals with a simple system, heterocyst formation in cyanobacteria filaments, yet complex enough to capture the main ingredients of some of the mechanisms for cell differentiation and pattern formation under external driving. The knowledge of the basic regulatory genes and their corresponding interactions allows for a detailed description of cell dynamics. We have derived the evolution equations of the involved genes based on the statistical mechanics of their corresponding regulatory processes. This allows us

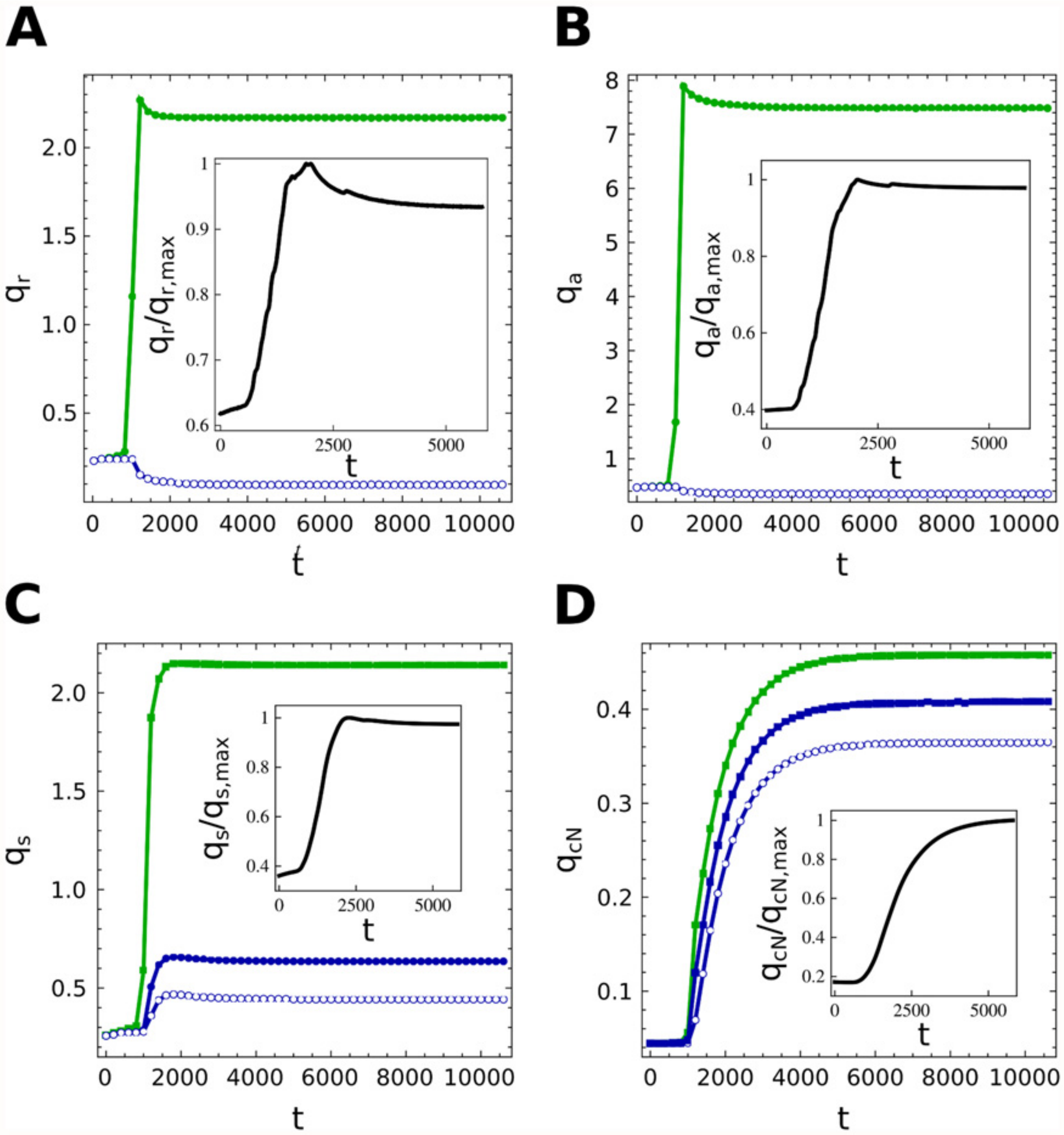

Fig 6. Time evolution of molecular concentrations. Time evolution of the main components of the differentiation in heterocysts (green) and vegetative cells (blue). Averages along the filament are also presented (black). Heterocysts, due to the early diffusion, evolve toward steady states of the type D of Fig. 5 characterized by high levels of HetR and NtcA while vegetative cells present very low concentrations of them (A and B). The levels of PatS and cN in vegetative cells depend on their distance to close heterocysts: C and D show the concentrations of PatS and cN in a heterocyst and in its first two neighbouring vegetative cells, which clearly highlight the effect of diffusion along the filament.

doi:10.1371/journal.pcbi.1004129.g006

to obtain a detailed description of the *continuous time dynamics* of the main regulatory proteins, in contrast to other discrete approximations based in boolean dynamics [15]. This kind of analysis has been shown to describe successfully other time dependent phenomena concerning cyanobacteria such as their circadian cycles [57]. The analysis of the unicellular dynamics has revealed that the two cellular stable states, vegetative and heterocyst cells, appear as attractors of the non-linear dynamics of the regulatory equations. However, the study of many coupled cells is needed as cyanobacteria do not differentiate when isolated.

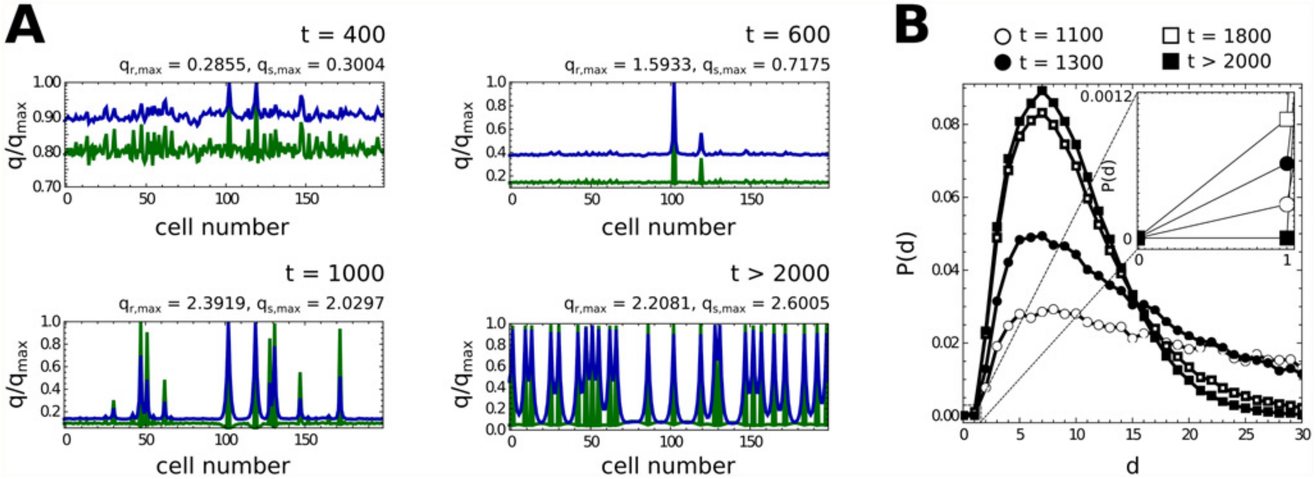

Fig 7. Heterocyst pattern. Time-evolution of the pattern of heterocysts (A) and of the probability distribution of the distance between consecutive heterocysts (B). Green and blue curves represent the concentration profiles of HetR and PatS (NtcA and cN are not presented since their behavior along the filament is comparable to that of HetR and PatS, see Fig. 6 to see the similarities). Small perturbations along the filament of vegetative cells (initially in the steady state B of Fig. 5) are amplified due to diffusion processes in a demonstration of Turing's theory [51]. New heterocysts appear in regions that are not dominated by the action of other heterocysts. Finally, the competition between nearby differentiating cells ceases the differentiation of some of them, as observed in B: consecutive heterocysts, which are created by strong perturbations, finally disapear due to the aforementioned competition. The final pattern presents localized levels of HetR (heterocysts) and a diffusive-like behavior of PatS, as expected.

doi:10.1371/journal.pcbi.1004129.g007

The model is rounded off by coupling a number of cells in a one-dimensional array so that combined nitrogen and PatS can diffuse along the cellular chain. We have shown that one important ingredient affecting the dynamical behavior of the chain is noise, which plays a key role in onset of the pattern formation, *i.e.*, the transition from the initial chain of vegetative cells to the steady state in which heterocysts coexist with vegetative cyanobacteria. Thus, the appearance of differentiation is, in our model, a pure stochastic event. The cooperative character of the filament is clear from the amount of noise needed to start the differentiation process which appears significantly smaller than that needed in isolated cells. The source of noise as well as its biological consequences is, nowadays a current topic of research [58]. In fact, at its initial state, differentiation of cells appears randomly along the filament, but shortly after its onset a characteristic distribution of heterocyst emerges. This distribution can be compared with the experimental one with a fairly good agreement [41].

Although the model presented here integrates both the internal cell dynamics and the coupling between cells via diffusion, there exist other ingredients that can be also incorporated. One issue that have not been considered in this work is the replication of vegetative cells. This effect has been taken into account in [14]. Although this improvement is relevant, it only affects, in our approach, to the mean separation between heterocysts, by opening a gap in the Γ-function shape of Fig. 7B and thus approaching better to the experimental distribution.

Other improvements to the approach presented here will come from the availability of more experimental data. Unlike other approaches [13] in which comparison is done (globally) with heterocyst distributions, our work would allow for a qualitative comparison of each component involved in the differentiation (see Fig. 6). Unfortunately, there is not enough experimental data to make a detailed fit so to extract reliable parameters. The availability of such data is extremely important both for having a better set of model parameters and to validate new models. A complete understanding of the mechanism that derive in phenotypic differentiation is the first step for a modular comprehension of the whole cell [59].

Methods

To reproduce the dynamics of Eq. (16) we make use of the integration scheme proposed in [54]. Eq. (16) is a set of stochastic differential equations (SDE) so its numerical integration requires generating a statistical representative trajectory for a discrete set of time-values. A SDE of the form

$$\dot{x} = f(x) + G(t), \tag{17}$$

where $G(t)$ is a Gaussian white noise with

$$\langle G(t) \rangle = 0, \text{ and, } \langle G(t)G(t') \rangle = \xi\delta(t' - t),$$

can be integrated through a Runge-Kutta integration algorithm by adding a particular Gaussian signal at each stage of the scheme. This algorithm coincides with the usual Runge-Kutta scheme for $\xi = 0$. In this work we have employed a $3_O 4_S 2_G$ algorithm, which is correct up to 3th order, is developed in 4 stages and uses 2 independent Gaussian random variables.

Supporting Information

S1 Text. Regulatory equations: a statistical mechanics approach.
(PDF)

S2 Text. Turing linear stability analysis.
(PDF)

Acknowledgments

We are grateful to S. Ares and J. Muñoz-García for sharing ideas and useful discussions. We also acknowledge the "Genetic Regulation and Physiology of Cyanobacteria" group at University of Zaragoza for sharing insight on the genetic of heterocyst formation.

Author Contributions

Conceived and designed the experiments: ATS JGG FF. Performed the experiments: ATS. Analyzed the data: ATS JGG FF. Contributed reagents/materials/analysis tools: ATS JGG FF. Wrote the paper: ATS JGG FF.

References

1. Koch A, Meinhardt H (1994) Biological pattern formation: from basic mechanisms to complex structures. Rev Mod Phys 66: 1481–1511. doi: 10.1103/RevModPhys.66.1481
2. Süel GM, Garcia-Ojalvo J, Liberman LM, Elowitz MB (2006) An excitable gene regulatory circuit induces transient cellular differentiation. Nature 440: 545–550. doi: 10.1038/nature04588 PMID: 16554821
3. Wolk C (1998) Heterocyst formation. Annu Rev Genet 30: 59–78. doi: 10.1038/nrmicro2242
4. Flores E, Herrero A (2010) Compartmentalized function through cell differentiation in filamentous cyanobacteria. Nat Rev Microbiol 8: 39–50, doi: 10.1146/annurev.genet.30.1.59
5. Schirrmeister B, Antonelli A, Bagheri H (2011) The origin of multicellularity in cyanobacteria. BMC Evol Biol 11: 45. doi: 10.1186/1471-2148-11-45 PMID: 21320320
6. Shi Y, Zhao W, Zhang W, Ye Z, Zhao J (2006) Regulation of intracellular free calcium concentration during heterocyst differenciation by HetR and NtcA in Anabaena sp. PCC7120. Proc Nat Acad Sci (USA) 103: 11334–11339. doi: 10.1073/pnas.0602839103
7. Toepel J, Welsh E, Summerfield T, Pakrasi H, Sherman L (2008) Differential Transcriptional Analysis of the Cyanobacterium Cyanothece sp. Strain ATCC 51142 during Light-Dark and Continuous-Light Growth. J Bacteriol 190: 3904–3913. doi: 10.1128/JB.00206-08 PMID: 18390663

8. Yoon H (1998) Heterocyst Pattern Formation Controlled by a Diffusible Peptide. Science 282: 935–938. doi: 10.1126/science.282.5390.935 PMID: 9794762

9. Zhang CC, Laurent S, Sakr S, Peng L, Bédu S (2006) Heterocyst differentiation and pattern formation in cyanobacteria: a chorus of signals. Mol microbiol 59: 367–375. doi: 10.1111/j.1365-2958.2005.04979.x PMID: 16390435

10. Mitchison GJ, Wilcox M (1972) Rule governing cell division in Anabaena. Nature 239: 110–111. doi: 10.1038/239110a0

11. Mitchison GJ, Wilcox M, Smith RJ (1976) Measurement of an inhibitory zone. Science 191: 866–868. doi: 10.1126/science.814620 PMID: 814620

12. Kumar K, Mella-Herrera R, Golden J (2010) Cyanobacterial heterocists. Cold Spring Harb Perspect Biol 2: a000315. doi: 10.1101/cshperspect.a000315 PMID: 20452939

13. Allard J, Hill A, Rutenberg A (2007) Heterocyst patterns without patterning proteins in cyanobacterial filaments. Dev Biol 312: 427–434. doi: 10.1016/j.ydbio.2007.09.045 PMID: 17976569

14. Brown A, Rutenberg A (2012) Reconciling cyanobacterial fixed-nitrogen distributions and transport experiments with quantitative modelling. Phys Biol 9: 016007. doi: 10.1088/1478-3975/9/1/016007 PMID: 22313598

15. Gedtzen Z, Salgado J, Osses A, Asenjo J, Rapaport I, Andrews B (2009) Modeling heterocyst pattern formation in cyanobacteria. BMC Bioinf 10: S16. doi: 10.1186/1471-2105-10-S6-S16

16. Laurent S, Chen H, Bédu S, Ziarelli F, Peng L, Zhang CC (2005) Nonmetabolizable analogue of 2-oxoglutarate elicits heterocyst differentiation under repressive conditions in Anabaena sp. PCC 7120. P Natl Acad Sci USA 102: 9907–9912. doi: 10.1073/pnas.0502337102

17. Muro-Pastor MI, Reyes JC, Florencio FJ (2001) Cyanobacteria perceive nitrogen status by sensing intracellular 2-oxoglutarate levels. J Biol Chem 276: 38320–38328. PMID: 11479309

18. Vázquez-Bermúdez MF, Herrero A, Flores E (2003) Carbon supply and 2-oxoglutarate effects on expression of nitrate reductase and nitrogen-regulated genes in Synechococcus sp. strain PCC 7942. FEMS Microbiol Lett 221: 155–159. doi: 10.1016/S0378-1097(03)00208-8 PMID: 12725920

19. Muro-Pastor MI, Reyes JC, Florencio FJ (2005) Ammonium assimilation in cyanobacteria. Photosynth Res 83: 135–150. doi: 10.1007/s11120-004-2082-7 PMID: 16143848

20. Wei TF, Ramasubramanian TS, Golden JW (1994) Anabaena sp. strain PCC 7120 ntcA gene required for growth on nitrate and heterocyst development. J Bacteriol 176: 4473–4482. PMID: 7913926

21. Frías JE, Flores E, Herrero A (1994) Requirement of the regulatory protein NtcA for the expression of nitrogen assimilation and heterocyst development genes in the cyanobacterium Anabaena sp. PCC 7120. Mol microbiol 14(4): 823–832. doi: 10.1111/j.1365-2958.1994.tb01318.x PMID: 7534371

22. Vázquez-Bermúdez MF, Herrero A, Flores E (2002) 2-Oxoglutarate increases the binding affinity of the NtcA (nitrogen control) transcription factor for the Synechococcus glnA promoter. FEBS lett 512: 71–74. doi: 10.1016/S0014-5793(02)02219-6 PMID: 11852054

23. Tanigawa R, Shirokane M, Maeda Si, Omata T, Tanaka K, Takahashi H (2002) Transcriptional activation of NtcA-dependent promoters of Synechococcus sp. PCC 7942 by 2-oxoglutarate in vitro. P Natl Acad Sci USA 99: 4251–4255. doi: 10.1073/pnas.072587199

24. Ramasubramanian TS, Wei TF, Oldham aK, Golden JW (1996) Transcription of the Anabaena sp. strain PCC 7120 ntcA gene: multiple transcripts and NtcA binding. J Bacteriol 178: 922–926. PMID: 8550535

25. Ramasubramanian TS, Wei TF, Golden JW (1994) Two Anabaena sp. strain PCC 7120 DNA-binding factors interact with vegetative cell- and heterocyst-specific genes. J Bacteriol 176: 1214–1223. PMID: 8113160

26. Buikema WJ, Haselkorn R (1991) Isolation and complementation of nitrogen fixation mutants of the cyanobacterium Anabaena sp. strain PCC 7120. J Bacteriol 173: 1879–1885. PMID: 1900504

27. Buikema WJ, Haselkorn R (1991) Characterization of a gene controlling heterocyst differentiation in the cyanobacterium Anabaena 7120. Gene Dev 5: 321–330. doi: 10.1101/gad.5.2.321 PMID: 1840555

28. Ehira S, Ohmori M (2006) NrrA directly regulates expression of hetR during heterocyst differentiation in the cyanobacterium Anabaena sp. strain PCC 7120. J Bacteriol 188: 8520–8525. doi: 10.1128/JB.01314-06 PMID: 17041048

29. Wisén S, Jiang F, Bergman B, Mannervik B (1999) Expression and purification of the transcription factor NtcA from the cyanobacterium Anabaena PCC 7120. Protein expres purif 17: 351–357. doi: 10.1006/prep.1999.1131

30. Alfonso M, Kirilovsky D (2001) Redox Control of ntcA Gene Expression in Synechocystis sp. PCC 6803. Nitrogen Availability and NtcA Protein 1. Plant Physiol 125: 969–981. doi: 10.1104/pp.125.2.969 PMID: 11161053

31. Li JH (2003) An increase in the level of 2-oxoglutarate promotes heterocyst development in the cyanobacterium Anabaena sp. strain PCC 7120. Microbiology 149: 3257–3263. doi: 10.1099/mic.0.26462-0 PMID: 14600238

32. Buikema WJ, Haselkorn R (2001) Expression of the Anabaena hetR gene from a copper-regulated promoter leads to heterocyst differentiation under repressing conditions. P Natl Acad Sci USA 98: 2729–2734. doi: 10.1073/pnas.051624898

33. Khudyakov IY, Golden JW (2004) Different functions of HetR, a master regulator of heterocyst differentiation in Anabaena sp. PCC 7120, can be separated by mutation. P Natl Acad Sci USA 101: 16040–16045. doi: 10.1073/pnas.0405572101

34. Zhou R, Wei X, Jiang N, Li H, Dong Y, Hsi KL, et al. (1998) Evidence that HetR protein is an unusual serine-type protease. P Natl Acad Sci USA 95: 4959–4963. doi: 10.1073/pnas.95.9.4959

35. Huang X, Dong Y, Zhao J (2004) HetR homodimer is a DNA-binding protein required for heterocyst differentiation, and the DNA-binding activity is inhibited by PatS. P Natl Acad Sci USA 101: 4848–4853. doi: 10.1073/pnas.0400429101

36. Muro-Pastor AM, Valladares A, Flores E, Herrero A (2002) Mutual dependence of the expression of the cell differentiation regulatory protein HetR and the global nitrogen regulator NtcA during heterocyst development. Mol microbiol 44: 1377–1385. doi: 10.1046/j.1365-2958.2002.02970.x PMID: 12068814

37. Valladares A, Muro-pastor AM, Herrero A, Flores E (2004) The NtcA-Dependent P 1 Promoter Is Utilized for glnA Expression in N 2—Fixing Heterocysts of Anabaena sp. Strain PCC 7120. J Bacteriol 186: 7337–7343. doi: 10.1128/JB.186.21.7337-7343.2004 PMID: 15489445

38. Fiedler G, Muro-Pastor A (2001) NtcA-Dependent Expression of the devBCAOperon, Encoding a Heterocyst-Specific ATP-Binding Cassette Transporter in Anabaena spp. J Bacteriol 183: 3795–3799. doi: 10.1128/JB.183.12.3795-3799.2001 PMID: 11371545

39. Hebbar PB, Curtis SE (2000) Characterization of devH, a gene encoding a putative DNA binding protein required for heterocyst function in Anabaena sp. strain PCC 7120. J Bacteriol 182: 3572–3581. doi: 10.1128/JB.182.12.3572-3581.2000 PMID: 10852891

40. Wu X, Liu D, Lee MH, James W, Golden JW (2004) patS Minigenes Inhibit Heterocyst Development of Anabaena sp. Strain PCC 7120 patS Minigenes Inhibit Heterocyst Development of Anabaena sp. Strain PCC 7120. J Bacteriol 186: 6422–6429. doi: 10.1128/JB.186.19.6422-6429.2004 PMID: 15375122

41. Yoon Hs, Golden JW (2001) PatS and Products of Nitrogen Fixation Control Heterocyst Pattern PatS and Products of Nitrogen Fixation Control Heterocyst Pattern. J Bacteriol 183: 2605–2613. doi: 10.1128/JB.183.8.2605-2613.2001 PMID: 11274121

42. Higa KC, Rajagopalan R, Risser DD, Rivers OS, Tom SK, Videau P, et al. (2012) The RGSGR amino acid motif of the intercellular signalling protein, HetN, is required for patterning of heterocysts in Anabaena sp. strain PCC 7120. Mol microbiol 83: 682–693. doi: 10.1111/j.1365-2958.2011.07949.x PMID: 22220907

43. Borthakur PB, Orozco CC, Young-Robbins SS, Haselkorn R, Callahan SM (2005) Inactivation of patS and hetN causes lethal levels of heterocyst differentiation in the filamentous cyanobacterium Anabaena sp. PCC 7120. Mol microbiol 57: 111–123. doi: 10.1111/j.1365-2958.2005.04678.x PMID: 15948953

44. Fay P (1992) Oxygen relations of nitrogen fixation in cyanobacteria. Microbiol Rev 56: 340–373. PMID: 1620069

45. Wolk CP, Austin S, Bortins J, Galonsky A (1974) Autoradiographic localization of ^{13}N after fixation of ^{13}N-labeled nitrogen gas by a heterocyst-forming blue-gree alga. J Cell Biol 61: 440–453. doi: 10.1083/jcb.61.2.440 PMID: 4208073

46. Buchler NE, Gerland U, Hwa T (2003) On schemes of combinatorial transcription logic. Proc Natl Acad Sci 100: 5136–5141. doi: 10.1073/pnas.0930314100 PMID: 12702751

47. Bintu L, Buchler N, Garcia H, Gerland U, Hwa T, Kondev J, et al. (2005) Transcriptional regulation by the numbers: models. Curr Opin Genet Dev 15: 116–124. doi: 10.1016/j.gde.2005.02.007 PMID: 15797194

48. Phillips R, Kondev J, Theriot J, Garcia H (2012) Physical Biology of the Cell. New York: Garland Science, second edition.

49. Manneville P (1990) Dissipative structures and weak turbulence. London: Academic Press.

50. Morelli LG, Uriu K, Ares S, Oates AC (2013) Computational Approaches to Developmental Patterning. Science 336: 187–191. doi: 10.1126/science.1215478

51. Turing AM (1952) The Chemical Basis of Morphogenesis. Philos Trans R Soc London Ser B 237: 37–62. doi: 10.1098/rstb.1952.0012

52. Meinhardt H (1982) Models of Biological Pattern Formation. London: Academic Press.

53. Murray JD (2003) Mathematical Biology II: Spatial Models and Biomedical Applications computational model predicts phenotype from genotype. New York: Springer-Verlag, third edition.

54. Greenside HS and Helfand E (1981) Numerical-Integration of Stochastic Differential Equations 2. Bell Syst Tech J 60: 1927–1940. doi: 10.1002/j.1538-7305.1981.tb00303.x

55. Mitchison GJ, Smith RJ, Road H (1973) Pattern formation in the blue-green alga, anabaena. I. Basic Mechanisms. J Cell Sci 12: 707–723. PMID: 4198321

56. Jang J, Shi L, Tan H, Janicki A, Zhang CC (2009) Mutual Regulation of ntcA and hetR during Heterocyst Differentiation Requires Two Similar PP2C-Type Protein Phosphatases, PrpJ1 and PrpJ2, in Anabaena sp Strain PCC 7120. J Bacteriol 191: 6059–6066. doi: 10.1128/JB.01271-08 PMID: 19633087

57. Teng SW, Mukherji S, Moffitt JR, de Buyl S, O?Shea EK (2013) Robust circadian oscillations in growing cyanobacteria require transcriptional feedback. Science 340: 737–740. doi: 10.1126/science.1230996 PMID: 23661759

58. Suel GM, Kulkarni RP, Dworkin J, Garcia-Ojalvo J, Elowitz MB (2007) Tunability and noise dependence in differentiation dynamics. Science 315: 1716–1719. doi: 10.1126/science.1137455 PMID: 17379809

59. Karr J, Sanghvi J, Macklin D, Gutschow M, Jacobs J, Bolival B, et al. (2012) A whole-cell computational model predicts phenotype from genotype. Cell 150: 389–401. doi: 10.1016/j.cell.2012.05.044 PMID: 22817898
